# Genome-wide development of transposable elements-based markers in foxtail millet and construction of an integrated database

**DOI:** 10.1093/dnares/dsu039

**Published:** 2014-11-26

**Authors:** Chandra Bhan Yadav, Venkata Suresh Bonthala, Mehanathan Muthamilarasan, Garima Pandey, Yusuf Khan, Manoj Prasad

**Affiliations:** National Institute of Plant Genome Research (NIPGR), Aruna Asaf Ali Marg, New Delhi 110 067, India

**Keywords:** foxtail millet (*Setaria italica* L.), transposable element-based markers, retrotransposons, DNA transposons, database

## Abstract

Transposable elements (TEs) are major components of plant genome and are reported to play significant roles in functional genome diversity and phenotypic variations. Several TEs are highly polymorphic for insert location in the genome and this facilitates development of TE-based markers for various genotyping purposes. Considering this, a genome-wide analysis was performed in the model plant foxtail millet. A total of 30,706 TEs were identified and classified as DNA transposons (24,386), full-length *Copia* type (1,038), partial or solo *Copia* type (10,118), full-length *Gypsy* type (1,570), partial or solo *Gypsy* type (23,293) and Long- and Short-Interspersed Nuclear Elements (3,659 and 53, respectively). Further, 20,278 TE-based markers were developed, namely Retrotransposon-Based Insertion Polymorphisms (4,801, ∼24%), Inter-Retrotransposon Amplified Polymorphisms (3,239, ∼16%), Repeat Junction Markers (4,451, ∼22%), Repeat Junction-Junction Markers (329, ∼2%), Insertion-Site-Based Polymorphisms (7,401, ∼36%) and Retrotransposon-Microsatellite Amplified Polymorphisms (57, 0.2%). A total of 134 Repeat Junction Markers were screened in 96 accessions of *Setaria italica* and 3 wild *Setaria* accessions of which 30 showed polymorphism. Moreover, an open access database for these developed resources was constructed (Foxtail millet Transposable Elements-based Marker Database; http://59.163.192.83/ltrdb/index.html). Taken together, this study would serve as a valuable resource for large-scale genotyping applications in foxtail millet and related grass species.

## 1. Introduction

Transposable elements (TEs) constitute a significant fraction of plant genomes and are considered to be one of the major forces driving genome evolution. Further, TEs are capable of changing its position in the genome through transposition and so they are called as ‘jumping genes’. Each transposition event generates new variability by creating mutations and altering the genome size of a cell. On the basis of their mode of replication and transposition, the TEs are categorized as Class I and Class II. The Class I includes retrotransposons that produce RNA intermediates which are copied into DNA and then inserted into new locations within the genome while Class II TEs are DNA transposons that move directly by a ‘cut and paste’ mechanism.^1^ Class I elements are further categorized into two subclasses, namely (i) LTR retrotransposons, flanked by long terminal repeats (LTRs), and (2) non-LTR elements which comprise Long-Interspersed Nuclear Elements (LINEs) and Short-Interspersed Nuclear Elements (SINEs). Retrotransposons are the most abundant mobile elements found in plant genomes,^2^ as the replicative mode of retroelement transposition enables the LTR retrotransposon to accrue high copy number. Indeed, in some grasses, LTR retrotransposons represent up to 90% of the genome.^2,3^ They constitute for >50% of the maize genome,^4,5^ 14% of the *Arabidopsis* genome^6^ and up to 90% of the wheat genome.^7^ Similar to other plants, grass genomes are also rich in repetitive elements derived from retrotransposons which get amplified themselves in the genome through an RNA-mediated retrotransposition process.

The wide distribution of TEs in the plant genome, abundance and their variable arrangement pattern among closely related species facilitates their use as informative marker to assess genetic diversity in plant breeding programmes. TEs-based marker system takes advantage of their transpositional activity by which they cause insertions and hence variations; and also, the presence of conserved domains facilitates designing of PCR primers. So far, five classes of TE junction-based markers have been developed which include Repeat Junction Markers (RJMs), Repeat Junction-Junction Markers (RJJMs), Insertion Site-Based Polymorphism (ISBP), Inter-Retrotransposon Amplified Polymorphism (IRAP) and Retrotransposon-Based Insertion Polymorphism (RBIP).^8^ Among these, RJMs are unique in the sense that cover both TE and gene region and hence can be useful in functional genomic studies. Development and utilization of very few insertional polymorphism-based markers were demonstrated in grass species. Wanjugi *et al.*^9^ exploited the unique and abundant TE insertion junction regions identified from diploid *Aegilops tauschii* to develop genome-specific repeat DNA junction markers (RJMs) for use in hexaploid wheat. Identification of repeat junctions and large-scale development of TE-based marker was also successfully performed in barley.^10^

Foxtail millet (*Setaria italica* L.) is a C_4_ Panicoid grass with smaller genome (∼515 Mb), in-breeding and short life cycle.^11,12^ These attributes along with its genetic close-relatedness to other millets, cereals and several biofuel crops have made foxtail millet a model crop.^13,14^ The release of draft genome sequence by BGI (Beijing Genomics Institute), China,^15^ and Joint Genome Institute (JGI) of the Department of Energy, USA,^16^ had expedited the high-throughput analysis of genome and large-scale development of genomic resources such as simple sequence repeats (SSRs),^17,18^ EST-derived SSRs^19^ and intron length polymorphic markers (ILPs).^20^ Considering the importance of foxtail millet, functional significance of TEs and the necessity of TE-based markers in genotyping applications, this study was performed to identify the different classes of TEs and develop molecular markers by utilizing the sequence information of TEs. Further, the developed resources are made available to the global research community through open access, web-based Foxtail millet Transposable Element-based Marker Database (FmTEMDb; http://59.163.192.83/ltrdb/index.html).

## 2. Materials and methods

### 2.1. Identification of full-length retrotransposons and estimation of insertion time

Genomic sequence of foxtail millet was retrieved from Phytozome (ftp://ftp.jgi-psf.org/pub/compgen/phytozome/v9.0/Sitalica/), and the full-length retrotransposons were predicted using LTR_FINDER tool (http://tlife.fudan.edu.cn/ltr_finder/).^21^ The same tool was used to identify the Target Site Repeat (TSR), Primer Binding Site (PBS) and Polypurine Tract (PPT), Integrase [IN (core) and IN (c-term)] and RNaseH (RH) region for each predicted retrotransposon. 3′ and 5′ LTRs were identified based on their start and end (TG and CA, respectively) using in-house Perl script with the following parameters; LTR sequence length is 100–3,500 bp, and maximum distance between LTRs is 10,000 bp.

The 3′ and 5′ LTR sequences of the same Copia- and Gypsy-type retrotransposons were aligned by ClustalW^22^ using default parameters, and the pairwise sequence divergence was calculated using the Ka/Ks calculator (https://code.google.com/p/kaks-calculator/wiki/KaKs_Calculator). Based on NG parameter model,^23^ the Ka and Ks, the numbers of synonymous (*S*) and non-synonymous (*N*) sites (*S* + *N* = *n*), and the numbers of synonymous (*S*_d_) and non-synonymous (*N*_d_) substitutions (*S*_d_ + *N*_d_ = *m*) were estimated. The time of insertion was calculated as described by Tamura *et al.*^24^

### 2.2. Identification of DNA transposons

DNA transposons were identified by RepeatMasker (http://www.repeatmasker.org/cgi-bin/WEBRepeatMasker) with reference to repeat databases including Maize Transposable Element Database (maize TEDB), TIGR Gramineae Repeats v2.0, TIGR *Triticum* Repeats v3.0, TIGR *Oryza* Repeats v3.3, TIGR *Hordeum* Repeats v3.0, TIGR Sorghum Repeats v3.0 and Triticeae repeat (TREP) sequence database.^8^ BLAST search was performed in RepeatMasker using default parameters with ‘do not mask simple repeats or low-complex DNA’ option to avoid the regions of low complexity, such as simple tandem repeats, polypurines and AT-rich regions that can lead to spurious matches in database searches.

### 2.3. Insertion of transposons into intronic regions and functional annotation of genes interrupted with TEs

The data of intronic regions were obtained from the gff file of *S. italica* available in Phytozome. The intronic sequences were further annotated for different classes of transposons, and nested TEs were predicted using RepeatMasker.^8^ Further, the genes interrupted with retrotransposons were annotated with the BLASTX algorithm using Blast2GO^25^ under expected value (*e*-value) of 1.0e−10 and minimal length cut-off value of 33 to exclude hits with minor local alignments. The Blast2GO annotation tool was used to assign the most probable Gene ontology (GO) terms to the genes, and the results were visualized by WEGO tool (Web Gene Ontology Annotation Plot).^26^

### 2.4. Transcriptional activation of TEs in various tissues of foxtail millet

To confirm the transcriptional activities of TEs in foxtail millet, the Illumina RNA-HiSeq data of four tissues, namely spica, stem, leaf and root were retrieved from European Nucleotide Archive [SRX128226 (spica); SRX128225 (stem); SRX128224 (leaf); SRX128223 (root)].^27^ The RNA-seq data were then filtered and mapped onto various classes of TEs using Bowtie 1.0.0 (http://bowtie-bio.sourceforge.net/index.shtml). The mapped reads were analysed in all the four tissues of foxtail millet.

### 2.5. Primer designing, PCR amplification and detection of polymorphisms

Unique repeat junctions in the foxtail millet TEs were identified by executing both BLASTN search and repeat junction finding algorithm of RJPrimers pipeline v1.0 (http://probes.pw.usda.gov/RJPrimers/). The fully annotated repeat databases chosen for executing BLASTN search and identification of repeat junctions were maize TEDB (1313 sequences), TIGR Gramineae Repeats v2.0 (2,942 sequences), TIGR *Triticum* Repeats v3.0 (452 sequences), TIGR *Oryza* Repeats v3.3 (21,807 sequences), TIGR *Hordeum* Repeats v3.0 (630 sequences), TIGR Sorghum Repeats v3.0 (120 sequences) and TREP sequence database. *E*-value cut-off was set to 1e−50 for the top hit and 1e−5 was used as maximum for all the hits to reduce the detection of false-positive repeat junctions. Primer3 tool was used for designing primer pairs for the repeat junctions (http://bioinfo.ut.ee/primer3-0.4.0/).

The primers were further validated in 96 *S. italica* accessions and 3 wild accessions (one each from *Setaria viridis*, *Setaria sphacelata* and *Setaria verticillata*) (Supplementary Table S1). Genomic DNA was extracted from the lyophilized tissue of young leaves as described by Pandey *et al.*^17^ The DNA was quantified using 0.8% agarose gel by comparing with λ-*Hin*dIII DNA (Fermentas) as marker. The PCR amplification reactions were performed in a 25 µl reaction volume containing 100 ng of genomic DNA, 1× Taq buffer, 2 mM of MgCl_2_, 0.2 mM dNTP mix (Promega), 0.5 mM each of the forward and reverse primers and four units of Taq polymerase (Biotools). The PCR reactions were performed in iCycler thermal cycler (Bio-Rad) and with one cycle of 3 min at 94°C, 34 cycles of 60 s at 94°C, 60 s at 60°C, 1.30 min at 72°C and a final extension of 10 min at 72°C. The PCR products were resolved on 2% agarose gel. The DNA bands were eluted from the gel using Real Genomics Hi Yield Gel/PCR Fragments Extraction Kit (Real Biotech Corporation) and cloned into pGEM^®^-T Easy vector (Promega) following the manufacturer's instructions. The recombinant plasmids were then transformed into *Escherichia coli* DH5α competent cells, and the plasmids were isolated from positive clones using *AccuPrep* Plasmid MiniPrep DNA Extraction Kit (Bioneer) following the manufacturer's protocol. The plasmids were sequenced in automated sequencer (3730xI DNA Analyzer, Applied Biosystems) using M13 forward and reverse primers. The sequence information was used to construct multiple sequence alignment using TARGeT-based multiple sequence aligner.^28^

### 2.6. Phylogenetic and Bayesian model-based population structure analysis

The marker profiles of 99 accessions of *Setaria* species were scored for the presence (1) or absence (0) of the amplicon and a binary matrix was generated. Co-migrating bands were assumed to be originated from the same genetic locus. Binary matrix was analysed using the DARwin software v5.0.158.^29^ Using pairwise similarity matrix of Jaccard's coefficient, the level of genetic diversity among the 99 accessions was calculated and an unweighted neighbour-joining (UNJ) tree was constructed with a bootstrap analysis of 1,000 replicates.^30^

The existence of a structure was assessed using STRUCTURE 2.3.3 software,^31^ based on Bayesian model-based cluster analysis. The method used 99 accessions of *Setaria* to infer the fraction of an individual accession's genetic ancestry that belongs to a population, for a given number of populations (*K*). The genotype of each individual accession is a function of the allele frequencies in the *K* populations (clusters) and the proportion of its genotype drawn from each of the *K* populations (q_k_). The ‘no admixture model’ was tested, as recommended for dominant loci and a permutation test using a Markov Chain Monte Carlo (MCMC) method was applied to examine the population structure. For each run, the burn-in time was 2,00,000, and the number of replications was 2,00,000.^32^ The MCMC chain was run six times, using a correlated allele frequency model (prior mean is 0.01, prior SD = 0.05 and Lambda set at 1.0 in the advance option of the STRUCTURE program). Since it was difficult to choose the ‘correct’ *K* from the Ln probability of data [Ln *P*(D)], the Δ*K* values were estimated as per the procedure suggested by Evanno *et al.*^33^ All the calculations pertaining to assignment of optimum *K* according to Evanno *et al.*^33^ were performed using Structure Harvester v0.9.94 software (http://taylor0.biology.ucla.edu/structureHarvester/). Maximum peak of ΔK was considered as true cluster number.

### 2.7. Comparative mapping of TEs of foxtail millet with related grass species

The foxtail millet TE sequences were BLASTN searched against the genomes of sorghum (*Sorghum bicolor*), maize (*Zea mays*), rice (*Oryza sativa*) and *Brachypodium distachyon* in Phytozome using default parameters. The hits with >80% similarity were taken, and the orthologues were confirmed by BLAST searching against the respective repeat databases. The comparative physical map was visualized using Circos v0.55 (http://circos.ca).^34^

### 2.8. Database construction

To facilitate wider usage of these annotated TEs and the respective markers, a web-based open access database was constructed using open source softwares (Apache, PHP and MySQL). The user friendly web interface allows easy access of the TEs and TE-based marker information such as the sequences of forward and reverse primers, its respective length, melting temperature (°C) and the status of wet-lab validation. Further, the CMap feature has been integrated in the database, which enables the user to visualize the physical map of the TEs and TE-based marker (either chromosome-wise or primer types-wise). The CMap interface also allows the user to visualize the comparative map of TEs between foxtail millet chromosomes and chromosomes of sorghum, maize, rice and *Brachypodium*.

## 3. Results and discussion

### 3.1. Identification of Class I TEs in foxtail millet

Class I TEs include retrotransposons that transpose via an RNA intermediate.^35^ Retrotransposons are divided into two major subclasses, namely LTR retrotransposons and the non-LTR retrotransposons, which differ in their structure and transposition cycle. LTRs are further classified into *Copia*-like and *Gypsy-*like, whereas non-LTRs are categorized as LINEs and SINEs. In foxtail millet, 2,608 intact full-length LTRs were predicted using LTR_FINDER tool. These LTRs were further analysed for the presence of coding regions such as ‘gag’ that encodes capsid-like protein, ‘pol’ encoding for protease, integrase and reverse transcriptase enzymes, and ‘env’ coding for envelope protein. In addition, the sequences from coding region of some retrotransposons were extracted using in-house Perl script and were confirmed with BLASTX analysis against the non-redundant database of NCBI.

**Figure 1.**
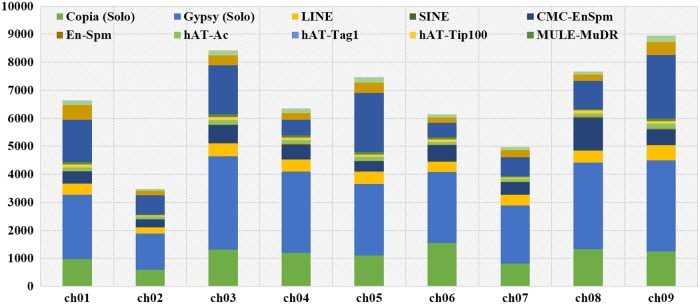
Distribution of different classes of TEs across the nine chromosomes of foxtail millet.

Of the 2,608 LTRs, 1,038 were found to be full-length *Copia* type and 1,570 were full-length *Gypsy* type (Supplementary Fig. S1; Supplementary Tables S2 and S3). In addition, partial or solo *Copia*-type (10,118) and *Gypsy*-type (23,293) retrotransposons were also identified (Fig. 1; Supplementary Tables S4 and S5). The length of *Copia* elements ranged from 1.4 to 23.9 kb with a mean of 7,708.36 bp, whereas the length of *Gypsy*-type LTRs varied from 1.8 to 25.9 kb with a mean of 11,776.3 bp. The full-length LTRs were defined by the presence of two LTRs (5′ and 3′; both starts and ends with TG and CA, respectively) flanking the coding regions, and PBS and PPT that vary depending on the TE family, ranging between 20 and 15 bp in length (Supplementary Fig. S2). Both the 3′ and 5′ LTR sequences of all LTR-type retrotransposons were extracted using in-house Perl script and analysed. The length of 3′ LTRs for *Copia*-type retrotransposons ranged from 0.1 to 3.4 kb with a mean length of 0.99 kb, whereas the length of *Gypsy*-type retrotransposons varied from 0.1 to 3.46 kb with a mean of 1.14 kb. Similarly, variations in length were also observed in 5′ LTRs of both *Copia*- and *Gypsy*-type retrotransposons. The length ranged from 0.1 to 3.4 kb with a mean length of 0.99 kb for *Copia* and 0.1 to 3.48 kb with a mean of 1.14 kb for *Gypsy* (Supplementary Tables S2 and S3).

The non-LTRs, distinguished from LTRs by the respective absence of LTRs, were categorized as LINEs and SINEs. A total of 3,653 LINEs and 53 SINEs were identified in foxtail millet genome (Supplementary Tables S6 and S7). Length of LINEs ranged from 0.1 to 14.0 kb with a mean length of 1.3 kb, while the length of SINEs varied from 0.1 to 1.8 kb with a mean length of 1.5 kb.

### 3.2. Chromosomal distribution of Class I TEs in foxtail millet

All the four subclasses of Class I TEs were evidenced to be distributed in all the nine chromosomes of foxtail millet (Fig. 1; Supplementary Fig. S2). A maximum of *Copia*-type TEs are present in chromosome 8 (∼14%; ∼3.6 TEs per Mb) and minimum in chromosome 7 (6.5%; ∼2 TEs per Mb). Average distribution of *Copia*-type TE in foxtail millet genome is 2.6 per Mb (Supplementary Tables S2 and S4). In case of *Gypsy*, a maximum of 199 elements were present in chromosome 4 (∼13%) and minimum in chromosome 7 (∼8%). Maximum density of *Gypsy*-type TEs was found in chromosome 6 (∼5 per Mb) and minimum was observed in chromosome 9 (∼3 per Mb) (Fig. 1; Supplementary Fig. S2; Supplementary Tables S3 and S5). Foxtail millet chromosome 9 comprised the maximum number of LINEs (532; 14.5%) and chromosome 2 had the minimum (233; ∼5%). The density of LINEs was maximum in chromosome 6 (10.5 per Mb) and minimum in chromosome 2 (∼5 per Mb) (Supplementary Table S6). In case of SINEs, maximum of these elements were observed in chromosome 11 (∼21%) and minimum in chromosome 7 (∼6%) (Fig. 1; Supplementary Table S7).

### 3.3. Estimation of LTR insertion time

Insertion time of LTRs in the foxtail millet genome was calculated to predict the time taken for the occurrence of each transposition events, which facilitates the movement of LTRs from one position to another in the genome. For *Copia*-type elements, the distribution of the divergence time ranged from 0.000767 to 3.93273 MYA (million years ago) with a mean value of 1.28 MYA (Supplementary Table S8). The distribution of the divergence time for *Gypsy*-type retrotransposons was estimated to be in a range of 0.000371–6.7687 MYA with a mean value of 0.82 (Supplementary Table S9). These results are in agreement with the previous studies where the insertion timings are reported in a range of 0.00–6.00 MYA.^36–38^ In rice, it was reported that 263 LTR-RTs (5%) have insertion dates <14,000 yrs old, which is approximately the time of rice domestication.^39–41^ Further, the Ka/Ks ratios estimated as >1 signified that LTRs have underwent positive selection. This positive selection at the DNA level could have resulted from the ability of TE sequences to replicate faster than the host genome.

### 3.4. Identification of Class II type TEs and its distribution in foxtail millet genome

A total of 24,386 DNA transposons belonging to Class II type TEs were identified in foxtail millet (Supplementary Table S10). The lengths of these TEs varied from 0.1 to 14.7 kb with a mean length of 0.78 kb. The 22,860 DNA transposons were further classified into 10 subclasses, namely DNA/CMC-EnSpm, DNA/En-Spm, DNA/hAT-Ac, DNA/hAT-Tag1, DNA/hAT-Tip100, DNA/MULE-MuDR, DNA/PIF-Harbinger, DNA/TcMar-Stowaway, RC/Helitron and DNA/Tourist based on similarity search with known TEs reported in other plant species. Of these, DNA/PIF-Harbinger accounts for the highest of Class II type TEs (12,758; ∼52%), followed by DNA/CMC-EnSpm (4,979; ∼20%). DNA/hAT-Tag1 (31) and DNA/Tourist (28) were found to be least in number, amounting for 0.1% of the Class II TEs identified in foxtail millet (Supplementary Table S10).

Chromosomal distribution data of these 24,386 DNA transposons revealed that a maximum of 3,901 were present in chromosome 9 (16%) and minimum in chromosome 2 (1,366; ∼28%) (Fig. 1). Higher density of Class II TEs was evidenced in chromosome 5 (∼71 per Mb) and lower in chromosome 2 (∼28 per Mb) with an average density of 61 DNA transposons per Mb (Supplementary Table S10).

### 3.5. Transcriptional activation of TEs in various tissues of foxtail millet

**Figure 2.**
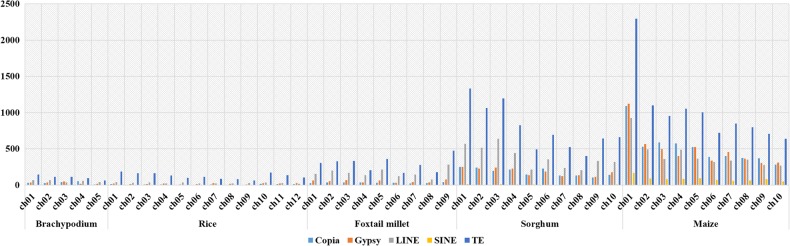
A comparative view of different classes of transposable elements in the intronic regions of *Brachypodium*, Rice, foxtail millet, Sorghum and maize genomes.

Retrotransposons were found to be transcriptionally active in all the four tissues, namely leaf, root, spica and stem (Fig. 2; Supplementary Tables S11–S15). A maximum of *Copia*-type TEs (∼80%) matched with RNA-HiSeq reads which revealed that *Copia*-type TEs were prevalently expressed in tissues of foxtail millet. Approximately 49% of *Gypsy*-type retrotransposons matched with RNA-HiSeq reads. Similarly, ∼10% LINEs, ∼15% SINEs and ∼16% DNA transposons matched with the expressed reads (Fig. 2; Supplementary Tables S11–S15).

### 3.6. Development of TE-based markers

All the 30,706 identified foxtail millet TEs were searched for potential repeat junctions (Supplementary Figs S3 and S4). Based on repeat junction, six types of primers were designed, namely retrotransposon-based insertion polymorphism (RBIP), IRAP, RJM, RJJM, ISBP and retrotransposon-microsatellite amplified polymorphism (REMAP) (Supplementary Tables S16–S21). Hence, a total of 20,278 primers were successfully designed, of which maximum was the ISBP (7,401; ∼36%) followed by RBIP (4,801; ∼24%). Only 57 primers could be designed for REMAP (∼0.2%). The numbers of IRAP, RJM and RJJM primers were 3,239 (∼16%), 4,451 (∼22%) and 329 (∼2%), respectively (Supplementary Tables S16–S21). Although these markers were expected to include the fragments between the TE sequence on one side and the TE-inserted unique gene sequence on the other, some of the primers were evidenced to show same conformation at different locations in foxtail millet genome. This generation of duplications is due to the RJPrimer tool, using which the TE-based markers were developed.

Although primers could not be designed for LINEs and SINEs using Primer3 because of the limitations in their lengths, three RJM primers were manually designed for LINEs by aligning foxtail millet CDS on genomic sequences using GeneSeqer tool.^42^ In addition, the TE-based forward and reverse primers were BLAST searched with available draft foxtail millet chromosomal pseudomolecule sequences to know their uniqueness/specificity in the foxtail genome. The results indicated that 1,522 (∼21%) of ISBP, 1,012 (∼21%) of RBIP, 712 (∼16%) of RJM, 546 (∼17%) of IRAP, 91 (∼28%) of RJJM and 8 (∼14%) of REMAP were unique.

### 3.7. Amplification and polymorphic potential of TE-based markers

**Figure 3.**
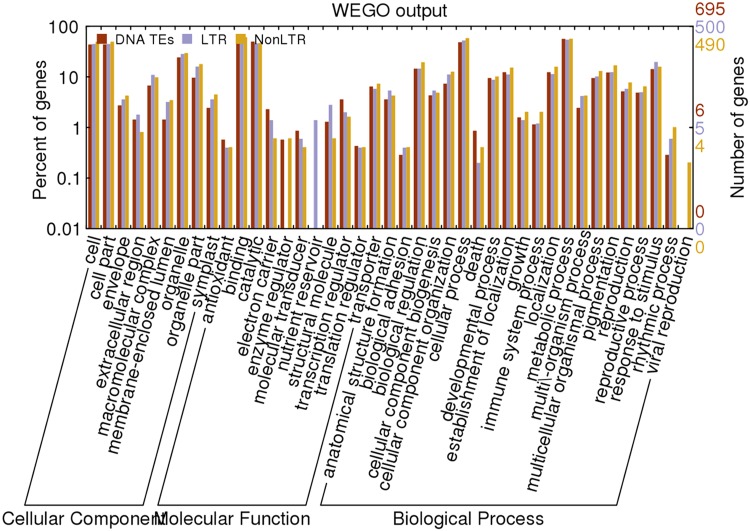
The relative frequencies of Gene Ontology (GO) hits assigned to the GO functional categories; Biological Process, Molecular Function, and Cellular Component for *Setaria italica* genes which were interrupted with transposable elements. ‘DNA TEs’ indicates DNA transposons; ‘LTR’ represents Copia and *Gypsy*-type retrotransposons and ‘NonLTR’ indicates LINEs and SINEs.

**Figure 4.**
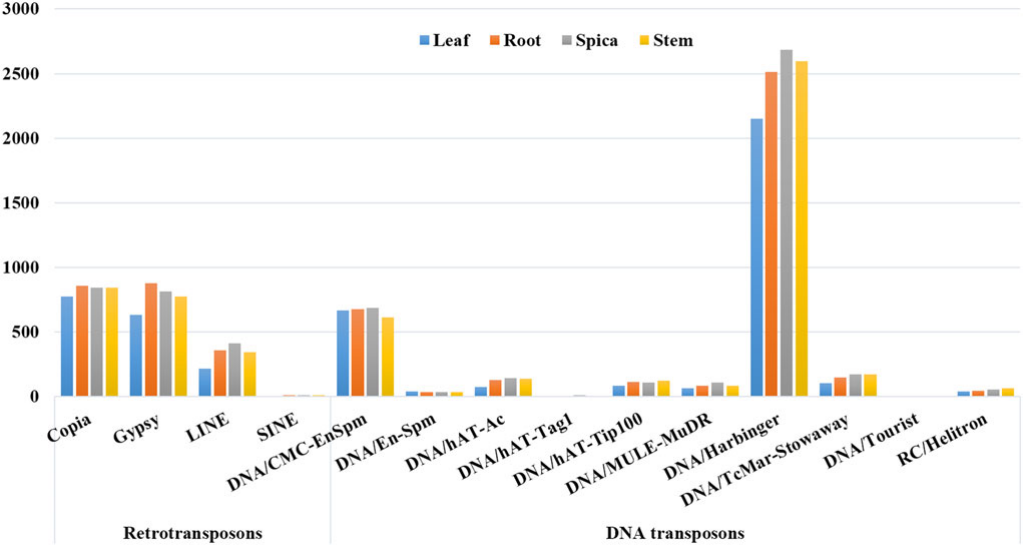
Frequency of transcriptionally active transposable elements present in four tissues of foxtail millet.

A total of 134 RJM primers were selected representing the nine chromosomes of foxtail millet for validation. Initially, all the 134 primer pairs were amplified in 96 accessions of *S. italica* and 3 wild *Setaria* (*S. viridis*, *S. sphacelata* and *S. verticillata*) accessions to examine the insertional polymorphism among these accessions (Fig. 3; Supplementary Table S22). Of these, 104 (∼78%) amplified unique single allele, whereas 30 primers (∼22%) amplified more than single allele and was evidenced to be polymorphic. The amplicons showing polymorphism were sequenced and compared with the reference genome available in Phytozome to confirm whether all the sequences of 30 polymorphic primer pairs show complete similarity with amplified sequences (Fig. 4; Supplementary Figs S6 and S7).

### 3.8. Identification of TEs present in intronic regions and functional annotation of genes interrupted with TEs

**Figure 5.**
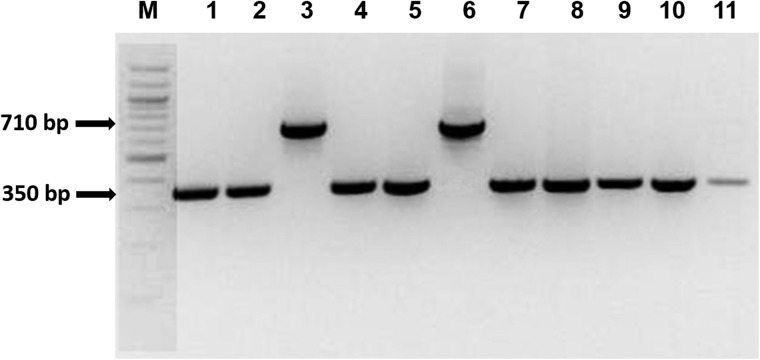
PCR amplification profile of Repeat Junction Marker ‘Solo_Gypsy_17547’. Lane M Marker; Lane 1: Foxtail millet cv. Prasad; Lane 2: cv. Lepakshi; Lane 3: IC403476; Lane 4: GS464; Lane 5: IC404178; Lane 6: IC403579; Lane 7: IC403476; Lane 8: IC403521; Lane 9: EC539248; Lane 10: EC539291; Lane 11: EC539300.

TEs are reported to play a crucial role in gene evolution by disrupting the genes. Upon getting integrated into the genome, TEs mediate chromosomal rearrangements, leading to accumulation of mutations and ultimately become transpositionally inactive. The presence of TEs was searched in the introns of 45,735 protein-coding genes of foxtail millet. Approximately 12% genes of foxtail millet were found to be integrated with TEs, of which ∼0.75% genes had *Copia*-type retrotransposons, ∼1% genes had *Gypsy* type, ∼3% genes had LINEs and ∼6% genes were interrupted with DNA transposons (Table 1). LINEs (1,497 interrupted genes) and DNA transposons (830 genes with PIF-Harbinger and 635 genes with TcMar-Stowaway) were observed to be predominantly integrated within genic region. Similar phenomenon of TE insertion in intronic region was also observed in sorghum, maize, rice and *Brachypodium* (Fig. 5; Table 1)*.* Further, the patterns of the nested TEs (the insertion of TEs into pre-existing TEs) for all the types of retrotransposons were analysed. A total of 4,927 inserted TEs were found to be inserted within 2,078 host TEs, thus revealing that >1 TE inserted into a single host TE. Of the total inserted TEs, 6.3% (308) was found to be inserted in RC/Helitron, 13.66% (673) in DNA/CMC-EnSpm, 11.26% (555) in DNA/En-Spm, 0.37% (18) in DNA/hAT-Ac, 0.30% (15) in DNA/MULE-MuDR, 0.41% (20) in DNA/TcMar-Stowaway, 3.75% (185) in DNA/PIF-Harbinger, 0.26%, (13) in DNA/Tourist, 0.52% (26) in LINE/L1, 26.91% (1,326) in Copia and 36.28% (1,788) in Gypsy (Supplementary Fig. S8). It has been reported that the movement of TEs in genomes results in the occurrence of nested TEs.^43^ These nested TEs in foxtail millet genome may negatively influence genome expansion and enrich the diversity of gene expression or regulation.

**Table 1.** Transposable elements interrupt the genes with clear signatures of insertions in intronic regions

TE types	ch01	ch02	ch03	ch04	ch05	ch06	ch07	ch08	ch09
Copia	25	38	34	37	33	31	22	27	42
Gypsy	62	54	70	36	63	33	43	36	77
LINE	154	199	168	138	212	122	144	76	284
SINE	4	3	1	4	3	0	0	2	3
CMC-EnSpm	29	26	36	16	21	16	24	28	41
En-Spm	0	2	1	0	0	0	1	0	3
hAT-Ac	6	9	5	8	10	6	11	8	11
hAT-Tag1	2	6	4	2	4	8	2	6	6
hAT-Tip100	8	4	6	7	7	4	3	1	3
MULE-MuDR	30	34	31	23	44	28	29	12	32
PIF-Harbinger	86	103	107	55	127	52	95	42	163
TcMar-Stowaway	82	89	80	42	83	27	66	42	124
RC/Helitron	59	54	62	49	62	27	45	37	89
Tourist	3	0	1	1	1	0	0	0	1
Total	305	327	333	203	359	168	276	176	473

**Figure 6.**
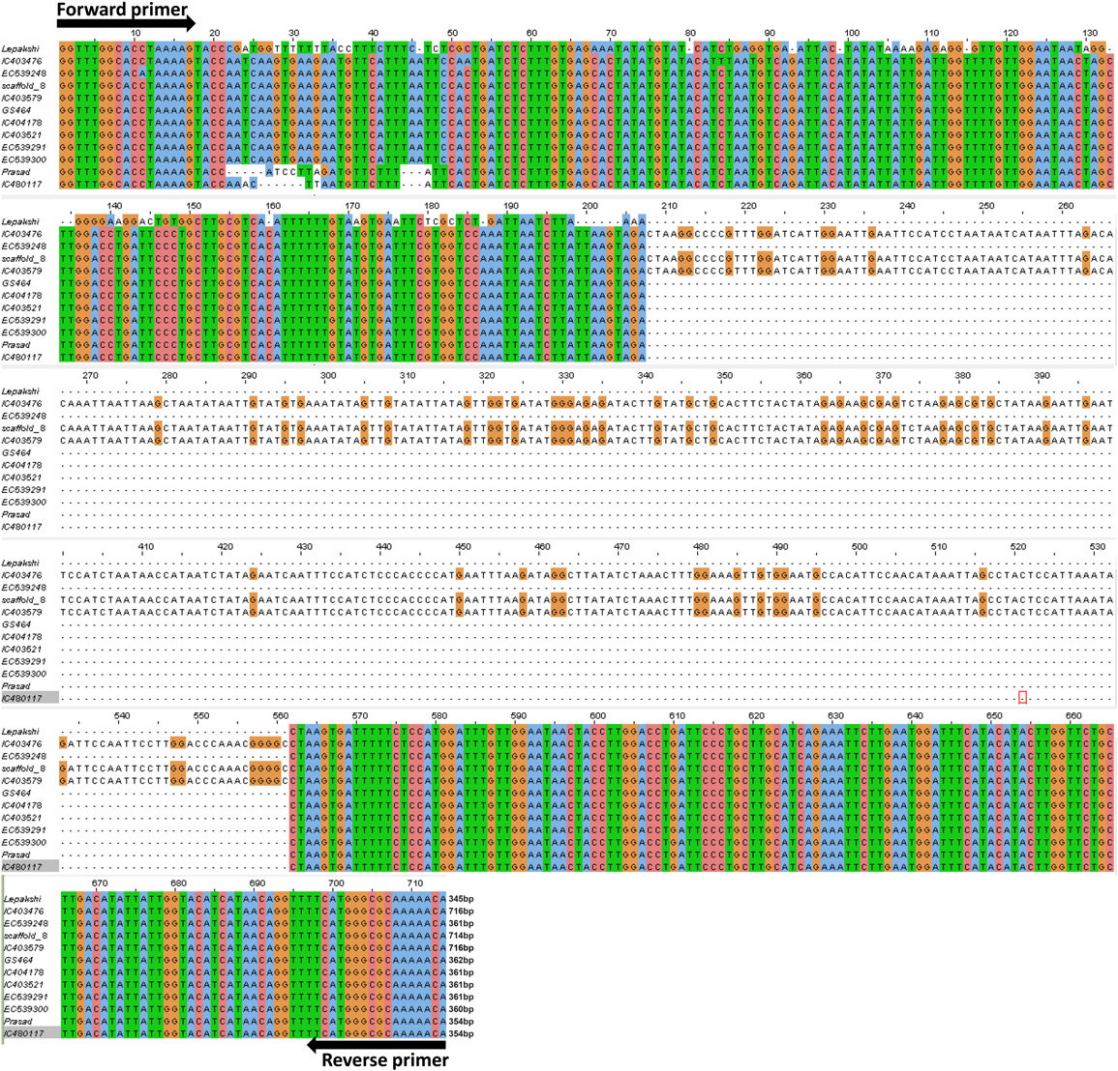
Multiple sequence alignment of different accessions of foxtail millet obtained from Repeat Junction Marker of ‘DNA13398’. ‘Scaffold_8’ denotes the transposable element sequence retrieved from the reference genome of foxtail millet available in Phytozome.

Hence, considering the role of TEs in regulation of gene expression, functional annotation of the genes interrupted with TEs was performed. The analysis revealed that predominant genes were involved in organ development (Fig. 6). Further chromatin regulatory genes containing SET domain gene family and AGO gene family which are the components of RNAi machinery were identified as the genes interrupted with TEs. Three genes namely RJM3, RJM4 and RJM7 that are interrupted with TEs were validated in *Setaria* species. Of these, RJM7 showed insertion of LINE-type transposons in 21st intron of EIF2ALPHA KINASE gene in *S. italica* as it acts as Eukaryotic translation initiation factor 2-alpha kinase (eif2-alpha kinase) in plants (Supplementary Fig. S7). However, there was no insertional signature observed in *S. verticillata*.

### 3.9. Phylogenetic and Bayesian model-based population structure analysis

To analyse the diversity among diverse germplasm of 99 *Setaria* accessions*,* a dendogram was generated from a similarity matrix using NJ clustering method. Unrooted NJ method resolved them into four major subgroups. Group 1 contains the largest number of individuals that were mostly originated from USA, whereas Group 2 comprises the accessions from India. Groups 3 and 4 predominantly comprise accessions from USA and China, respectively (Supplementary Fig. S9).

The TE-based marker data of 99 accessions of *Setaria* were also analyzed for the genetic structure. The software was run for the number of fixed subgroups (*K*) from 2 to 12, and more than three runs were performed for each *K* (Supplementary Table S23) and Δ*K* was estimated. Structure Harvester software based on Evanno method has delineated the optimum number of *K* as 4 (Supplementary Fig. S10; Supplementary Table S24). Similar to Hierarchical analysis, Bayesian model-based cluster analysis revealed that the 99 individuals were clustered into four groups, A–D (*K* = 4) (Supplementary Fig. S11). These individuals were further classified into the ones with ‘pure’ ancestry (where >80% of their inferred ancestry was derived from only one of the clusters) and ‘mixed ancestry’ or ‘admixtures’ (where >20% of inferred ancestry was derived from more than one cluster). Majority of the accessions (76) belonged to the ‘pure’ ancestry (Supplementary Table S25). The remaining accessions were of ‘mixed’ ancestry. A comparison of the results from Bayesian model-based STRUCTURE analysis with the NJ-based tree revealed considerable congruence. Two out of the four STRUCTURE-based clusters matched with the specific groups of the NJ-based tree. However, Clusters 2 and 3 were the exceptions, where the accessions from NJ-based group were not contributed to correspondence group. By understanding the ISBP due to RJM, analysing the evolutionary aspects is possible using phylogenetic approach. These markers were also proved successful in analysing genetic diversity analysis and construction of physical and genetic linkage maps in wheat.^9^ The major advantage of RJM is that it indicates the insertion polymorphism where different allelic states (the presence and absence of the transposon insertion) at a locus are revealed.^44^ Because of this unique advantage, RJMs are used in genetic, physical and radiation mapping studies.^9^

### 3.10. TE-based comparative mapping between foxtail millet and related grass species

**Figure 7.**
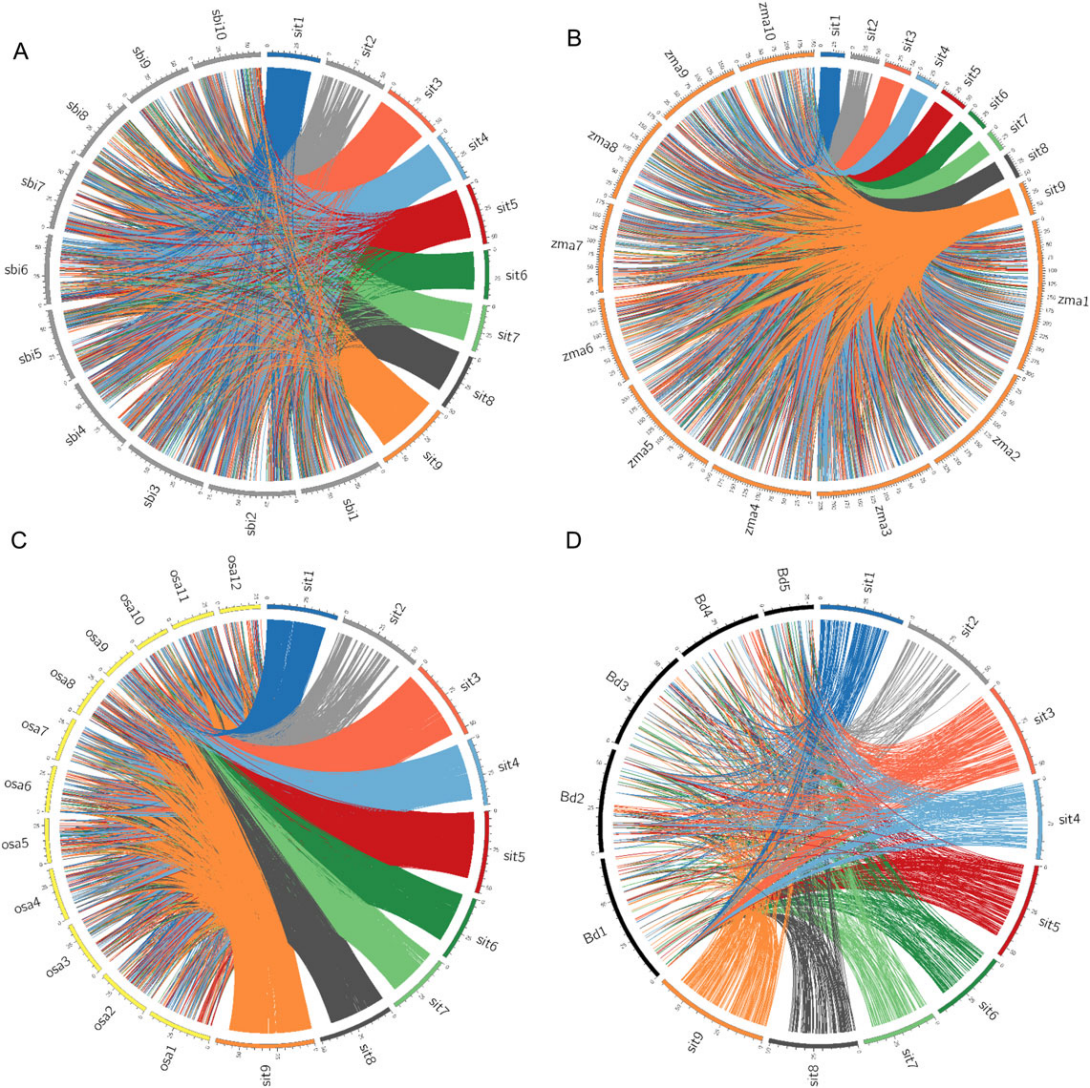
Comparative physical map of foxtail millet TEs with (A) sorghum, (B) maize, (C) rice and (D) *Brachypodium*.

TE-based comparative orthologous relationships between 30,706 foxtail millet TEs and TEs of sorghum, maize, rice and *Brachypodium* were analysed (Fig. 7; Supplementary Tables S26–S29). Of the 30,706 TEs, 14,008 (∼46%) showed maximum synteny with sorghum, 12,485 (∼40%) with maize, 9,634 (∼31%) with rice and 1,313 (∼4%) with *Brachypodium*. The data revealed a decrease in the degree of synteny with respect to increase in the phylogenetic distance. Interestingly, TEs mapped in foxtail millet chromosome 9 showed highest synteny with all the four grass species [2,419 (∼17%) with sorghum, 2,103 (∼17%) with maize, 1,666 (∼17%) with rice and 205 (∼16%) with *Brachypodium*] (Fig. 7). Similarly, TEs mapped in chromosome 2 of foxtail millet showed minimum synteny with all the four grasses [777 (∼5%) with sorghum, 715 (∼6%) with maize, 510 (∼5%) with rice and 69 (∼5%) with *Brachypodium*] (Supplementary Tables S26–S29). The wider genetic distances and low syntenic relationships among foxtail millet and other monocot genomes based on TE-based markers could be explained either through low conservation of TEs and/or species-specific transpositions. The independent evolutionary and divergence patterns of TEs have led to evolve unique transposition patterns in diverse crop lineages for generation of species-specific TEs resulting in their low conservation and synteny. This TE-based comparative mapping provides insights on the TEs in sorghum, maize, rice and *Brachypodium* and would enable map-based isolation and analysis of TEs in these grass species.

### 3.11. Online web-resource implementation and user interface

**Figure 8.**
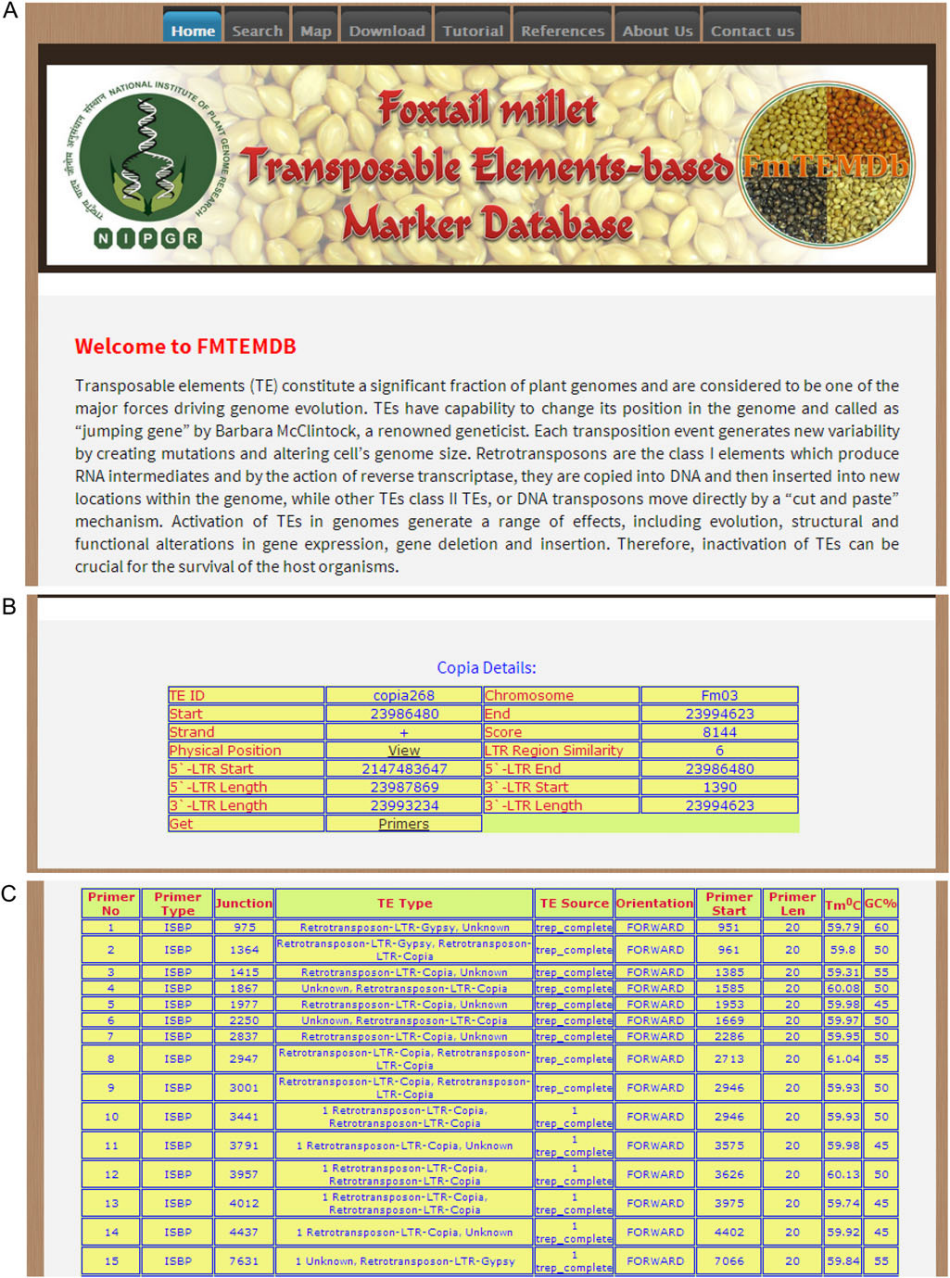
Screenshots of foxtail millet Transposable Elements-based Marker Database. (A) Home page, (B) the details of a *Copia*-type retrotransposon displayed and (C) details of primers present in a *Copia*-type retrotransposon.

**Figure 9.**
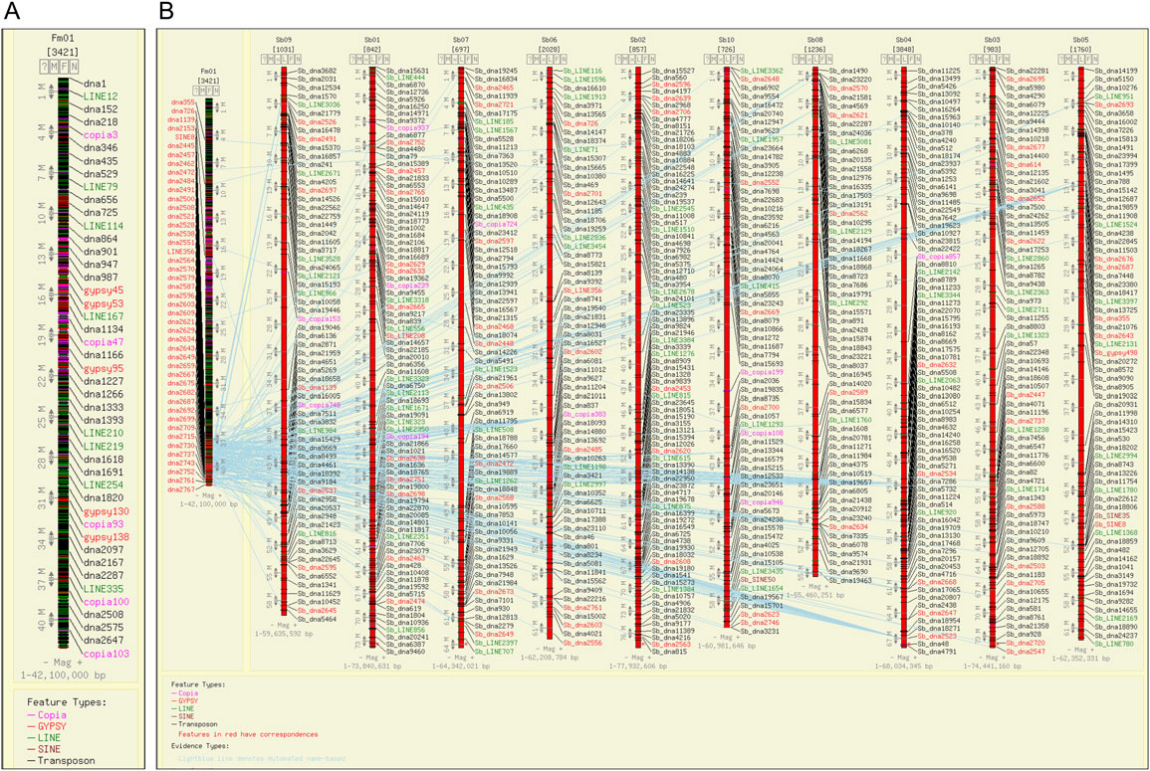
Screenshots of CMap interface of foxtail millet Transposable Elements-based Marker Database. (A) Physical map of foxtail millet chromosome 1 showing all the transposable elements; (B) comparative mapping of transposable elements between chromosome 1 of foxtail millet and all the 10 chromosomes of sorghum. The blue lines indicate the syntenic partners.

Using the three-level schema of Apache, PHP and MySQL, open access Foxtail millet Transposable Element-based Marker Database (FmTEMDb; http://59.163.192.83/ltrdb/index.html) was constructed (Fig. 8). The database is a repository of complete TE data along with respective marker information. The details of TEs can be searched using six different search criteria such as TE IDs, type of TE, chromosome, etc. For each TE, the database will provide preliminary information of TE ID, chromosomal location, orientation of the coding strand, subclass (if any) and hyperlinks to retrieve the primers and view the physical map. Under primer details, the database will display the primer type, junction, TE type, TE source, strand orientation, start and end position of primers, melting temperature and GC percentage. The CMap feature of FmTEMDb allows the interactive visualization of physical and comparative map of TEs (Fig. 9). The map could be browsed either by type of TE or chromosome-wise. Further, all the data stored in the database are available for download. Although the database is user friendly, a tutorial is also provided (Supplementary Fig. S12).

## 4. Conclusions

TEs are one of the major components of the plant genome, and they are reported to play a crucial role in functional genome diversity and phenotypic variations. Hence, analysing the organization of TEs in the genome would enable the researchers in dissecting the interplay between TEs and nearby gene expression which would enhance understanding the role of TEs in shaping the crop phenotypic diversity. The advent of next-generation sequencing and high-throughput sequence analysis platforms had facilitated the whole-genome sequencing and analysis of important crop plants. With the availability of genome sequence information, it is possible to investigate the organization of TEs in the genome. Further, the polymorphic potential of several types of TEs such as retrotransposons had encouraged the development of TE-based molecular markers that are useful for high-throughput genotyping applications.^45^ The major cause of genome evolution is because of the TEs, which has generated genetic diversity upon which selection can act. Further, TE transposition is also an important factor for nucleotide-base mutation rate, and thus, TEs serve as potential agents of evolutionary changes. TEs also induce phenotypic changes associated with domestication or diversification of cultivated plants. It also causes gene disruption by creating insertion or deletion in exon or intron region, which could be the major force towards the differential expression and regulation of gene.

Foxtail millet is a model crop for studying the genetics and genomics of several millets, cereals and bioenergy grasses.^13,14^ Hence, identifying the TEs, classifying and analysing its organization, and developing TE-based molecular markers in foxtail millet would serve as an important resource for millets, cereals and bioenergy genomics. Considering this, the present study was performed to identify a total of 30,706 TEs in foxtail millet and is classified into respective classes and subclasses. Further, the TEs present in intronic regions were identified, and functional annotation of respective genes was performed. Using the RNA-sequence data of four tissues, the transcriptional activation of TEs was analysed, and comparative physical mapping of foxtail millet TEs with sorghum, maize, rice and *Brachypodium* was performed. From 30,706 TEs, 20,278 markers were developed which belonged to six types. Of these, 134 RJMs were screened in 96 accessions of *S. italica* and 3 wild *Setaria* accessions of which 30 showed polymorphism. To provide the developed TE information to the global science community, a web-based, open access database (FmTEMDb; http://59.163.192.83/ltrdb/index.html) was constructed. Promisingly, the TE data of foxtail millet along with the large-scale marker information reported in this study will be a valuable resource for foxtail millet genomic studies including genomic selection, fine mapping and phylogenetic analysis. Further, this would also assist in gaining new insights on the genome structure of this model crop as well as the potential of TEs in genetic variation studies.

## Supplementary data

Supplementary data are available at www.dnaresearch.oxfordjournals.org.

## Funding

The authors' work in this area was supported by the core grant of National Institute of Plant Genome Research (NIPGR), New Delhi, India. Funding to pay the Open Access publication charges for this article was provided by the National Institute of Plant Genome Research, New Delhi, India.

## Supplementary Material

Supplementary Data
