# Supplementary material for: Genome-wide development of transposable elements-based markers in foxtail millet and construction of an integrated database
Source: DNA Res. 2014 Nov 26;22(1):79–90. doi: 10.1093/dnares/dsu039 (PMC4379977; doi:10.1093/dnares/dsu039)
Supplement: Supplementary Data [file supp_22_1_79__index.html]

Genome-wide development of transposable elements-based markers in foxtail millet and construction of an integrated database — Supplementary Data 

# Genome-wide development of transposable elements-based markers in foxtail millet and construction of an integrated database

## Supplementary Data

Supplementary Data

**Files in this Data Supplement:**

- Supplementary Data - Pdf file
- Supplementary Table 1 - xls file
- Supplementary Table 2 - xls file
- Supplementary Table 3 - xls file
- Supplementary Table 4 - xls file
- Supplementary Table 5 - xls file
- Supplementary Table 6 - xls file
- Supplementary Table 7 - xls file
- Supplementary Table 8 - xls file
- Supplementary Table 9 - xls file
- Supplementary Table 10 - xls file
- Supplementary Table 11 - xls file
- Supplementary Table 12 - xls file
- Supplementary Table 13 - xls file
- Supplementary Table 14 - xls file
- Supplementary Table 15 - xls file
- Supplementary Table 16 - xls file
- Supplementary Table 17 - xls file
- Supplementary Table 18 - xls file
- Supplementary Table 19 - xls file
- Supplementary Table 20 - xls file
- Supplementary Table 21 - xls file
- Supplementary Table 22 - xls file
- Supplementary Table 23 - xls file
- Supplementary Table 24 - doc file
- Supplementary Table 25 - xls file
- Supplementary Table 26 - xls file
- Supplementary Table 27 - xls file
- Supplementary Table 28 - xls file
- Supplementary Table 29 - xls file
