# Supplementary material for: Genome-wide development of transposable elements-based markers in foxtail millet and construction of an integrated database
Source: DNA Res. 2014 Nov 26;22(1):79–90. doi: 10.1093/dnares/dsu039 (PMC4379977; doi:10.1093/dnares/dsu039)
Supplement: Supplementary Data [file supp_dsu039_dsu039supp.pdf]

Supplementary materials

Supplementary Figure S1: Distribution of full length LTR-type retrotransposons across the nine chromosomes of foxtail millet.

Supplementary Figure S2: Genome-wide prediction of Full length LTR retrotransposon from foxtail genome using LTR finder software.

Supplementary Figure S3: BLAST analysis showing unique junction region for designing the junction based primer.

Supplementary Figure S4: BLAST analysis showing repetitive region in the sequence (highlighted red in color) to demarcate the junction site. Arrow shows one primer from junction site and other primer from other region of the sequence.

Supplementary Figure S5: PCR amplification profile of Repeat Junction Marker (a) dna13398, (b) dna16027, (c) dna19096 and (d) LINE2650. The detailed information for 24 accessions (SN. 25-48) of *Setaria italica* are listed in Supplementary Table S1.

Supplementary Figure S6: Multiple sequence alignment of 88 accessions of *Setaria italica* accessions along with reference genome amplified with LINE2650 primer. The detailed information for 88 accessions of *Setaria italica* are listed in Supplementary Table S1.

Supplementary Figure S7: Multiple sequence alignment of 11 accessions of *Setaria italica* accessions along with reference genome amplified with RJM7 primer. The detailed information for 11 accessions (RJM07:reference genome; IC480117, IC403579, cv. Prasad, cv. Lepakshi, IC403476, GS464, IC404178, IC403521, EC539248, EC539291) of *Setaria italica* are listed in Supplementary Table S1.

Supplementary Figure S8: Distribution of different types of nested TE subclasses.

Supplementary Figure S9: An un-rooted neighbor-joining tree for the 99 individuals of *Setaria italica* alongwith three wild species.

Supplementary Figure S10: Graphical representation showed the detection of the true number of groups *K** resulted after structure analysis.

Supplementary Figure S11: Bar plot of *Setaria italica* of 99 accessions were generated by STRUCTURE 2.3.4 using the admixture model with correlated allele frequencies. Groups are represented by colours.

Supplementary Figure S12: Tutorial of Foxtail millet Transposable Element-based Marker Database.

Supplementary Table S1: List of plant materials used in the present study

Supplementary Table S2: Details of *Copia*-type retrotransposons identified in foxtail millet

Supplementary Table S3: Details of *Gypsy*-type retrotransposons identified in foxtail millet

Supplementary Table S4: Details of partially or solo *Copia*-type retrotransposons identified in foxtail millet

Supplementary Table S5: Details of partially or solo *Gypsy*-type retrotransposons identified in foxtail millet

Supplementary Table S6: Details of *LINEs* (Non-LTR type) identified in foxtail millet

Supplementary Table S7: Details of *SINE*s (Non-LTR type) identified in foxtail millet

Supplementary Table S8: Estimation of insertion time of *Copia*-type transposable elements

Supplementary Table S9: Estimation of insertion time of *Gypsy*-type transposable elements

Supplementary Table S10: Details of DNA transposons identified in foxtail millet

Supplementary Table S11: Presence of transcriptionally active *Copia*-type retrotransposons in various tissues of foxtail millet

Supplementary Table S12: Presence of transcriptionally active *Gypsy*-type retrotransposons in various tissues of foxtail millet

Supplementary Table S13: Presence of transcriptionally active non-LTR type retrotransposons (LINE) in various tissues of foxtail millet

Supplementary Table S14: Presence of transcriptionally active non-LTR type retrotransposons (SINE) in various tissues of foxtail millet

Supplementary Table S15: Presence of transcriptionally active DNA transposons in various tissues of foxtail millet

Supplementary Table S16: Details of RBIP primers designed in foxtail millet

Supplementary Table S17: Details of IRAP primers designed in foxtail millet

Supplementary Table S18: Details of RJM primers designed in foxtail millet

Supplementary Table S19: Details of RJJM primers designed in foxtail millet

Supplementary Table S20: Details of ISBP primers designed in foxtail millet

Supplementary Table S21: Details of REMAP primers designed in foxtail millet

Supplementary Table S22: Primer sequences of 134 transposable elements junction based markers validated in foxtail millet

Supplementary Table S23: The raw STRUCTURE output used for determination of true number of K

Supplementary Table S24: Determination of K using Evanno’s method

Supplementary Table S25 Summary of distribution of pure ancestral *Setaria italica* individuals

Supplementary Table S26: Syntenic relationships between transposable elements of foxtail millet and sorghum

Supplementary Table S27: Syntenic relationships between transposable elements of foxtail millet and maize

Supplementary Table S28: Syntenic relationships between transposable elements of foxtail millet and rice

Supplementary Table S29: Syntenic relationships between transposable elements of foxtail millet and *Brachypodium*

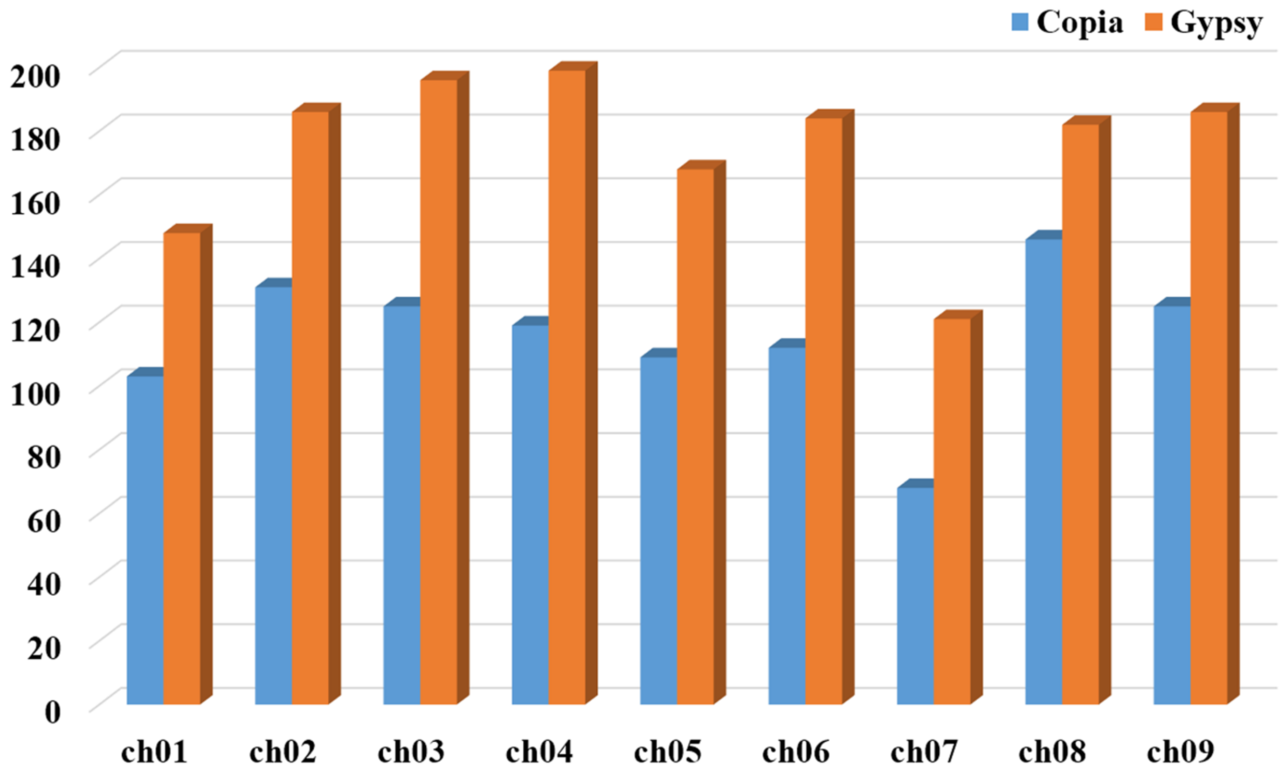
Copia
Gypsy
200
180
160
140
120
100
80
60
40
20
0
ch01
ch02
ch03
ch04
ch05
ch06
ch07
ch08
ch09

```
Score     : 7 [LTR region similarity:0.993]
Status    : 11111011000
5'-LTR    : 37113223 - 37114414 Len: 1192
3'-LTR    : 37126275 - 37127466 Len: 1192
5'-TG     : TG , TG
3'-CA     : CA , CA
TSR       : 37113218 - 37113222 , 37127467 - 37127471 [ACGGG]
Sharpness: 0.5,0.529
Strand + :
PPT   : [13/15] 37126216 - 37126230
Domain: 37117399 - 37117935 [possible ORF:37117198-37118769, (RT)]
Details of exact match pairs:
37126275-37126345[71] (1) 37126347-37126366[20] (1) 37126368-37126397[30] (12) 37126410-37126482[73] (6) 37126489-37126694[206]
37113223-37113293[71] (1) 37113295-37113314[20] (1) 37113316-37113345[30] (12) 37113358-37113430[73] (6) 37113437-37113642[206]
Details of the LTR alignment(5'-end):
                                               |37126275
GGAGGAGCACTCCTGCTCCACCACAGGCTCGGGGGCTACTGTTGGGGGAAATATTAACGATCCCCTAAGACAACATTTAA
|| | | | ||| || | ||          ||||       |*||||||||||||||||||||||||||||||||||||||
GG-GTAACGCTCATG-T-CA---------CGGG------TGTTGGGGGAAATATTAACGATCCCCTAAGACAACATTTAA
                   *---------****------|37113223
Details of the LTR alignment(3'-end):
                                37127466|*****
TTACTAGTCTAAGTCTTGATCCGCTGATCTTGACAACGACAACGGGTTT-CTA--T------CC----CGC-TGC-TCTTC
||||||||||||||||||||||||||||||||||||||*||    ||    |||  |       ||    ||| ||| ||| |
TTACTAGTCTAAGTCTTGATCCGCTGATCTTGACAACGACAGTTGGCGCGCTAGGTAGGGGGCCTTTGCGCGTGCATCTCC
                                37114414|
Details of PPT(+):
AAACAGCGAAAGAAG
|37126216
```

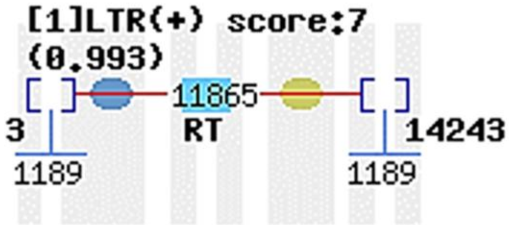

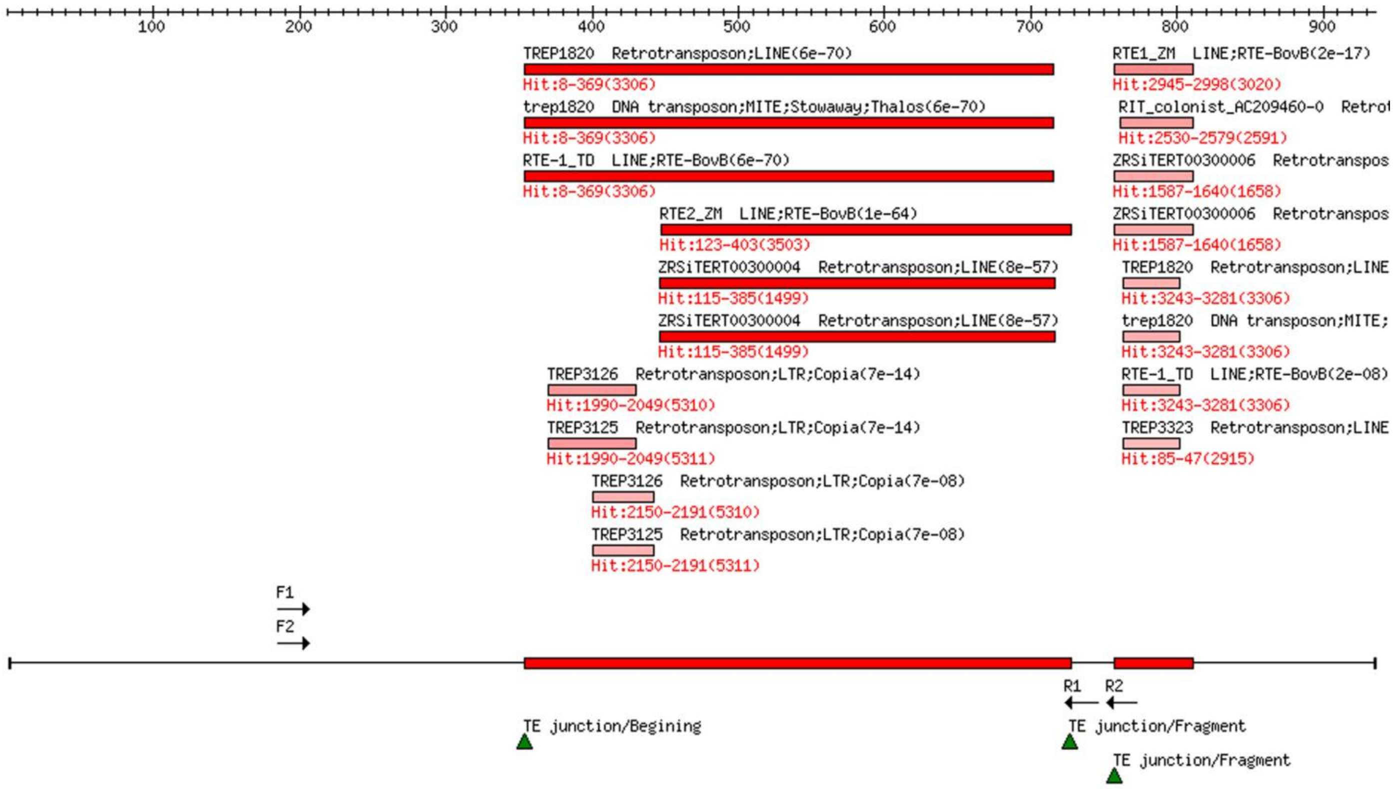
100
200
300
400
500
600
700
800
900
TREP1820 Retrotransposon;LINE(6e-70)
Hit:8-369(3306)
trep1820 DNA transposon;MITE;Stowaway;Thalos(6e-70)
Hit:8-369(3306)
RTE-1_TD LINE;RTE-BovB(6e-70)
Hit:8-369(3306)
RTE2_ZM LINE;RTE-BovB(1e-64)
Hit:123-403(3503)
ZRSiTERT00300004 Retrotransposon;LINE(8e-57)
Hit:115-385(1499)
ZRSiTERT00300004 Retrotransposon;LINE(8e-57)
Hit:115-385(1499)
TREP3126 Retrotransposon;LTR;Copia(7e-14)
Hit:1990-2049(5310)
TREP3125 Retrotransposon;LTR;Copia(7e-14)
Hit:1990-2049(5311)
TREP3126 Retrotransposon;LTR;Copia(7e-08)
Hit:2150-2191(5310)
TREP3125 Retrotransposon;LTR;Copia(7e-08)
Hit:2150-2191(5311)
RTE1_ZM LINE;RTE-BovB(2e-17)
Hit:2945-2998(3020)
RIT_colonist_AC209460-0 Retrot
Hit:2530-2579(2591)
ZRSiTERT00300006 Retrotranspos
Hit:1587-1640(1658)
ZRSiTERT00300006 Retrotranspos
Hit:1587-1640(1658)
TREP1820 Retrotransposon;LINE
Hit:3243-3281(3306)
trep1820 DNA transposon;MITE;
Hit:3243-3281(3306)
RTE-1_TD LINE;RTE-BovB(2e-08)
Hit:3243-3281(3306)
TREP3323 Retrotransposon;LINE
Hit:85-47(2915)
F1
F2
R1
R2
TE junction/Begining
TE junction/Fragment
TE junction/Fragment

1	GTACAAGAAGCATTTAGGTAGTGATCCACCGATCCATTATAGTGCACTATCAAGGAGTAGCTTTATAACTTTTTTTGTTTATAGGAGCATCTTTGATAGCACTACAATGTGGAAATTGCAACTCACACACTTGGATTCGTGGAATAAGTT
151	ATCTTCCGTGTGACATCGTGTCTCGTTAGATAAATGATCATGCTTCTCCACACCCAACCAACCATATCAAAAGGCCAACCAAAAGATTCGATATGGACTTGTAGAACTATGCTACTGTGGTTATAAATGCTTTTTCTTGGGAATATTTTA
	>>>>>>>>>>>>>>>>>>>>
	>>>>>>>>>>>>>>>>>>>>
301	GTTTCTATTTAAGTGGTTGTGCTAGATTATTATATAGATTATTATGAACTCTACCTATGTTGTCTCAGTATAGCAGGTCCTAAGCCCTGGCAAAGGAGGAGGGTTGTGATAGGCTTGGTGAGCCAACGTTAAACCTAGCCATTGTAATGG
451	AGATGAAACCCAAGAGAAATCCGTTGGGGCGTAACCCTCTTAGCGACGCGCCATATCGGAACCCGTGTATGGTGTTAAATGGGCAAGGGCCGGGTCGTCTCCCCCTTGGTGACGTGTCGTGTCGCGTTTTGAGAATGGTGTCAAGTGAGC
601	AAGGATCGGGTCGTCATTTCCTTAGTGGCACGCTACATCGACGCCCGGGTGTAGTGAAAAATGAGCAAGGGTCTTCACATCTCTCTCGACGGGTGCGAAGGGCAAGGAAGCTAGTCGAACCAACTAGTTTATTTTGCCCGAACCCTGATT
	<<<<<<<<<<<<<<<<<<<<<
751	TGTGGCTCTTGTTGGGTTTCAATTCTAGCCTACCCCAACTTGCTTGGGACTGAAAGGCTATGTTGTTGTTGTTGTTGTTGTTGTTGTTGCTAGATTATTATGGGTATGAACATCATTTAATGCAAATGTAGCATTTTGCTAATAGTT
	<<<<<<<<<<<<<<<<<<<<
901	TTGATGACATCAGCTAGTTTATCGAATGCTTCAGG

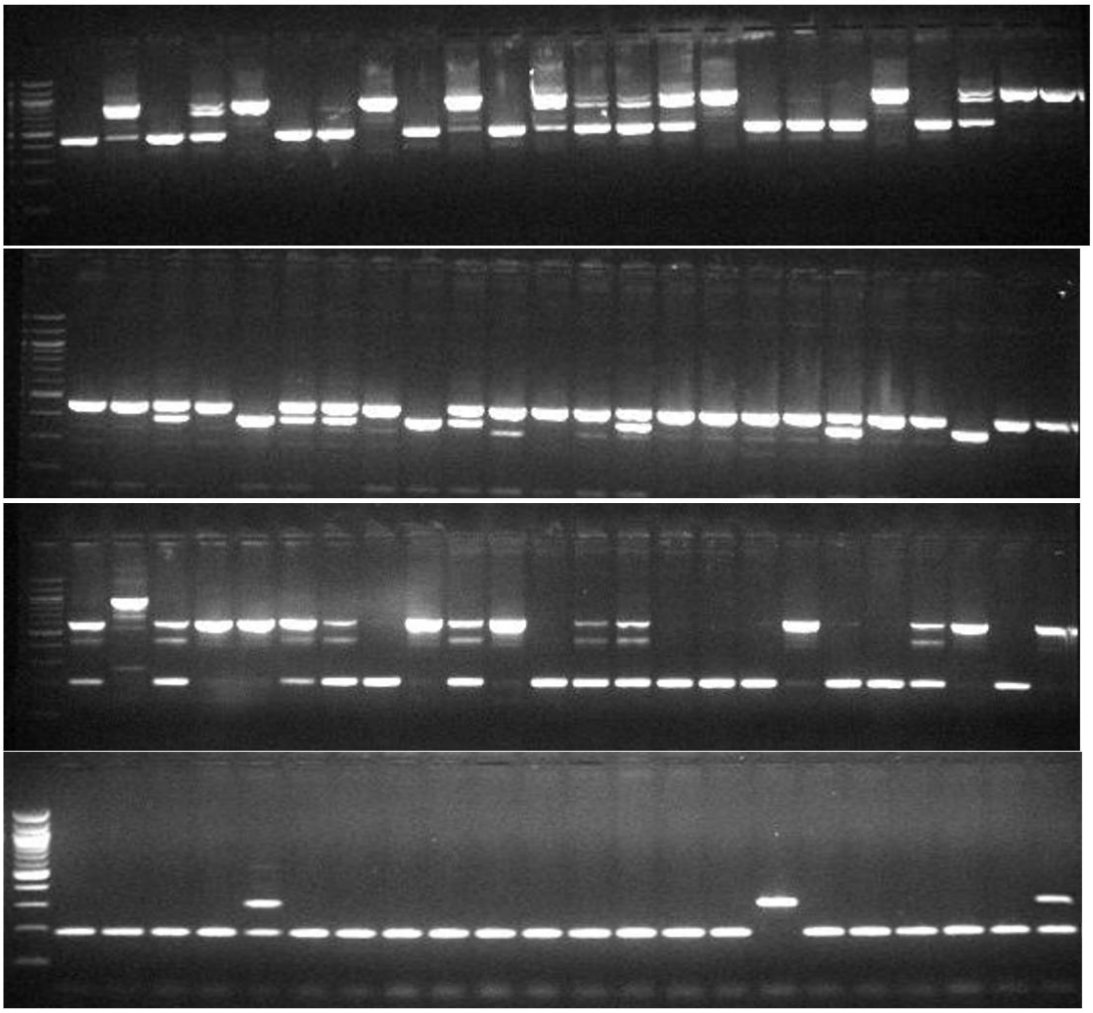

(a) dna13398

(b) dna16027

(c) dna19096

(d) LINE2650

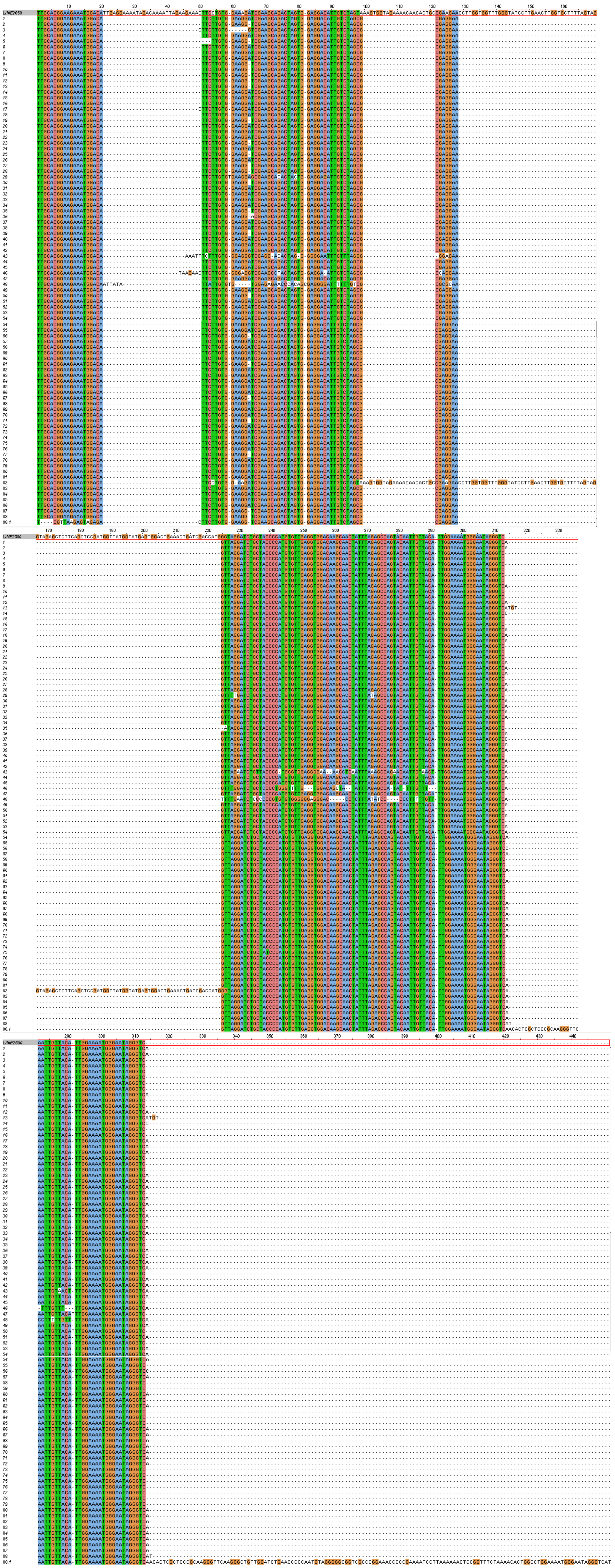
LINE2650

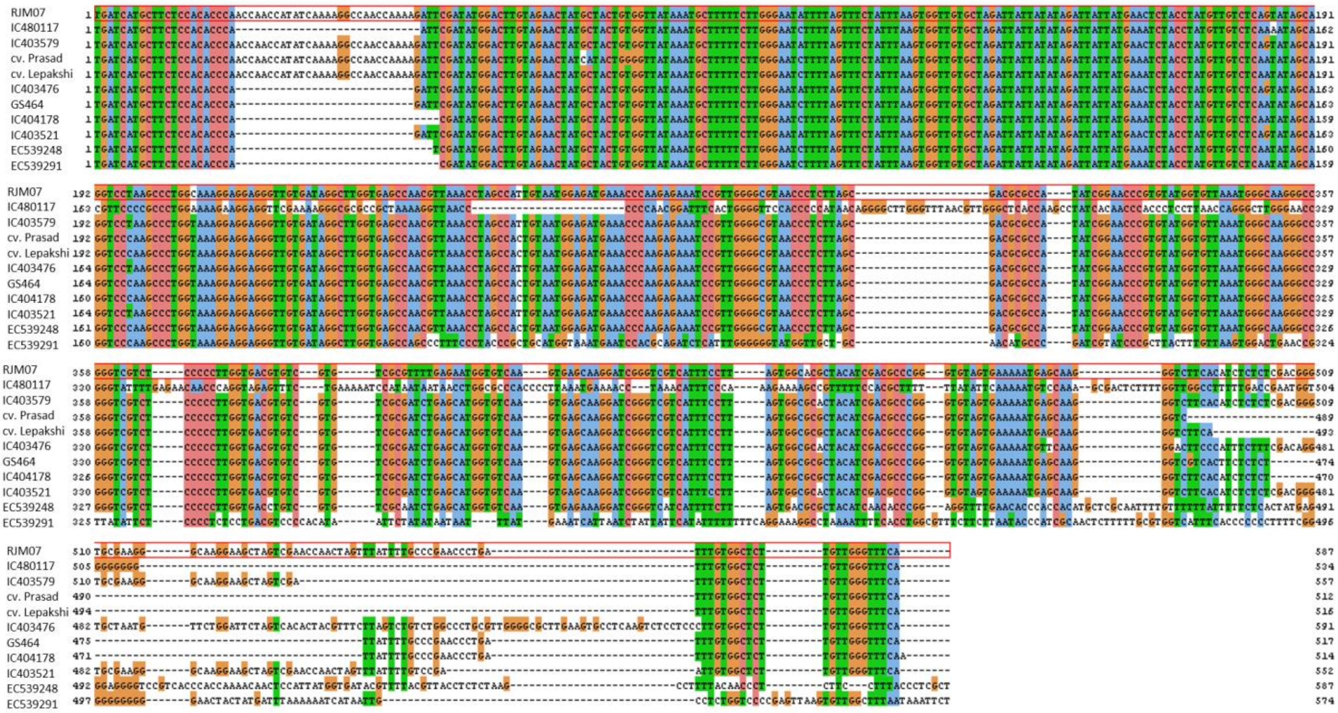
RJM07
IC480117
IC403579
cv. Prasad
cv. Lepakshi
IC403476
GS464
IC404178
IC403521
EC539248
EC539291

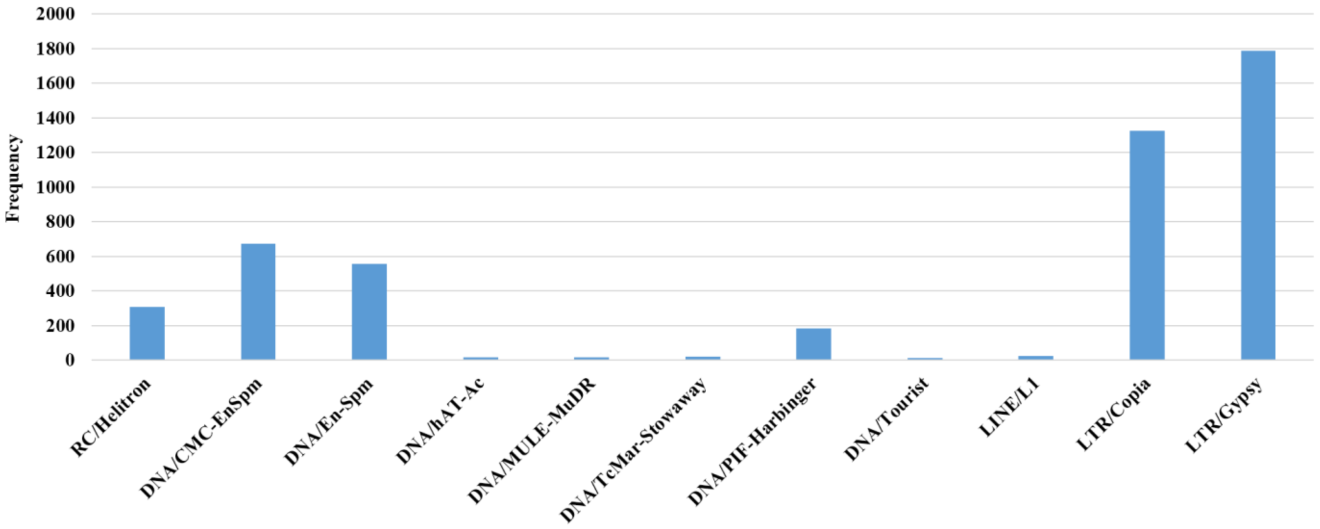

Frequency
2000
1800
1600
1400
1200
1000
800
600
400
200
0
RC/Helitron
DNA/CMC-EnSpm
DNA/En-Spm
DNA/hAT-Ac
DNA/MULE-MuDR
DNA/TcMar-Stowaway
DNA/PIF-Harbinger
DNA/Tourist
LINE/L1
LTR/Copia
LTR/Gypsy

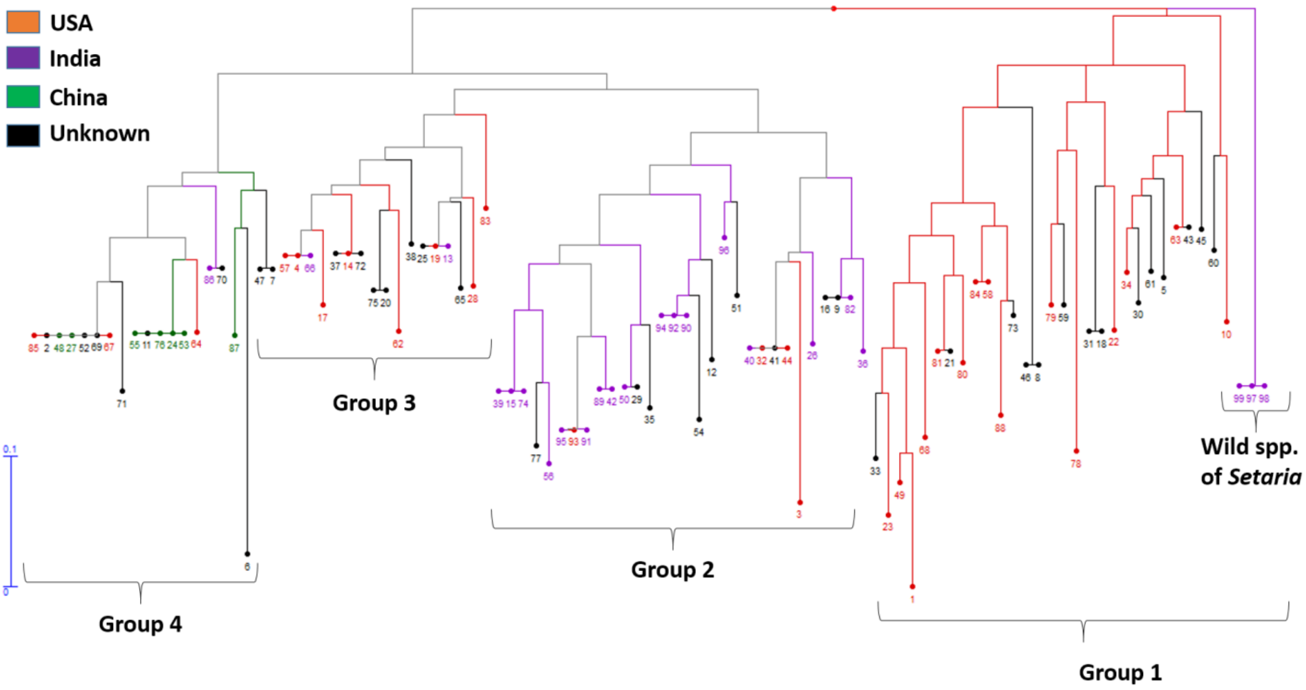
USA
India
China
Unknown
0.1
0
Group 4
Group 3
Group 2
Group 1
Wild spp. of *Setaria*

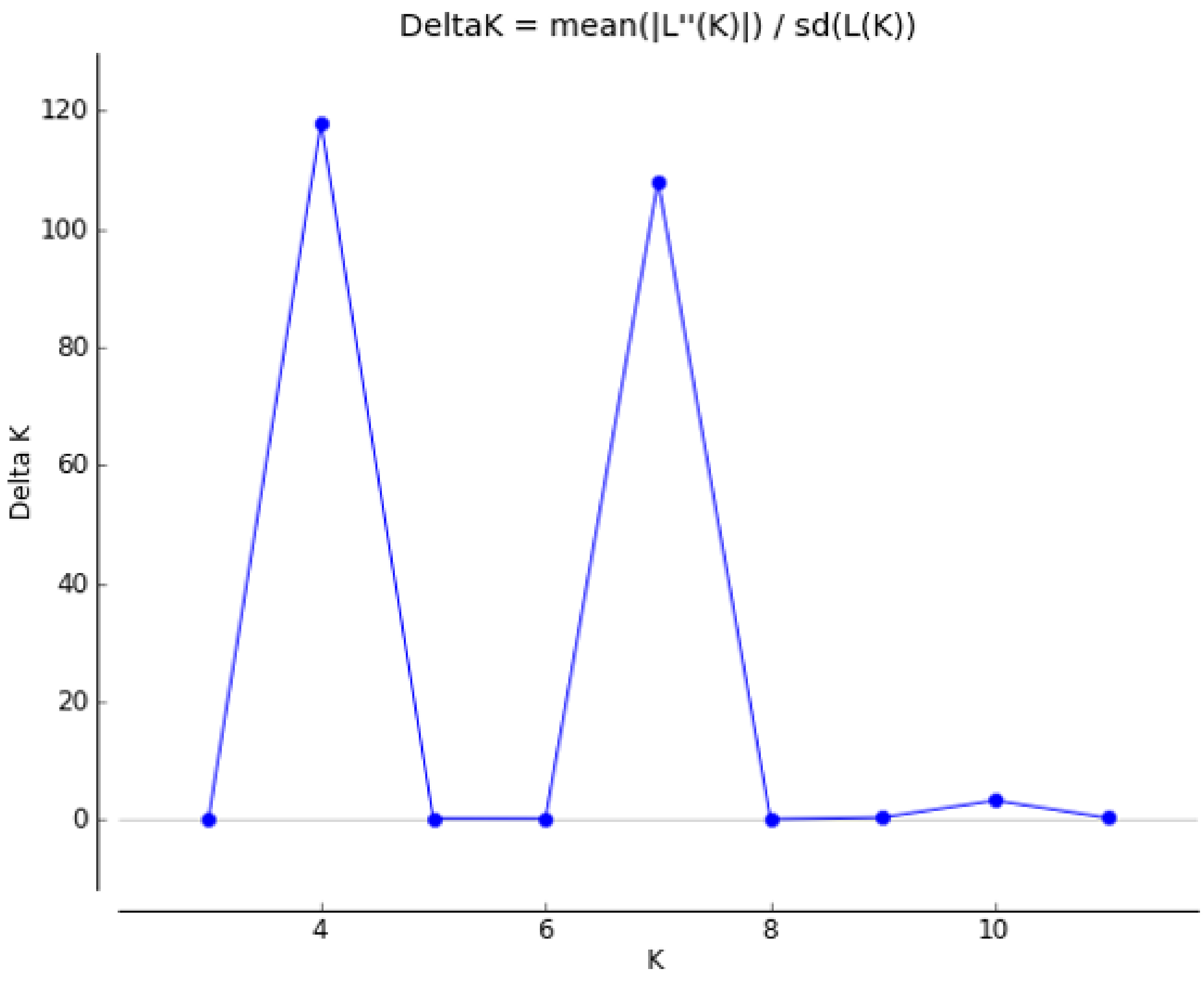
DeltaK = mean(|L''(K)|) / sd(L(K))
Delta K
120
100
80
60
40
20
0
4
6
8
10
K

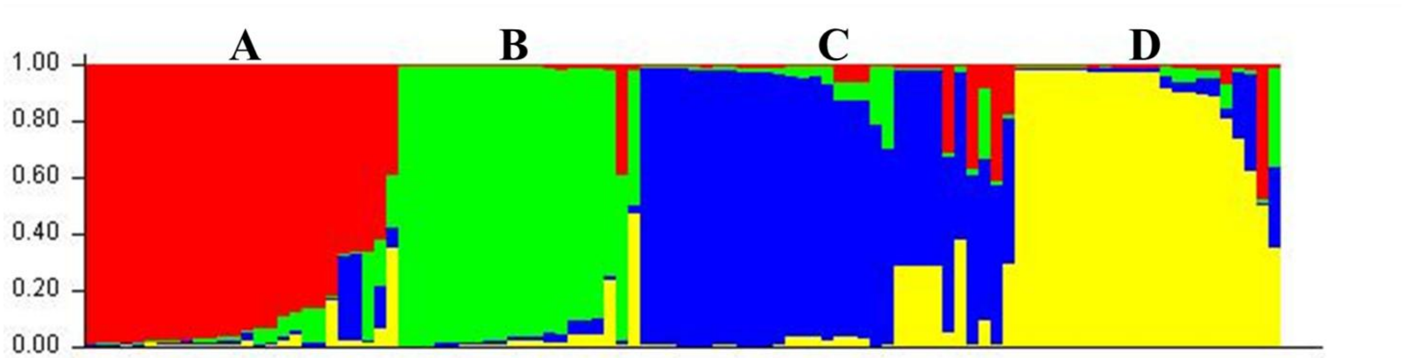
A
B
C
D
1.00
0.80
0.60
0.40
0.20
0.00

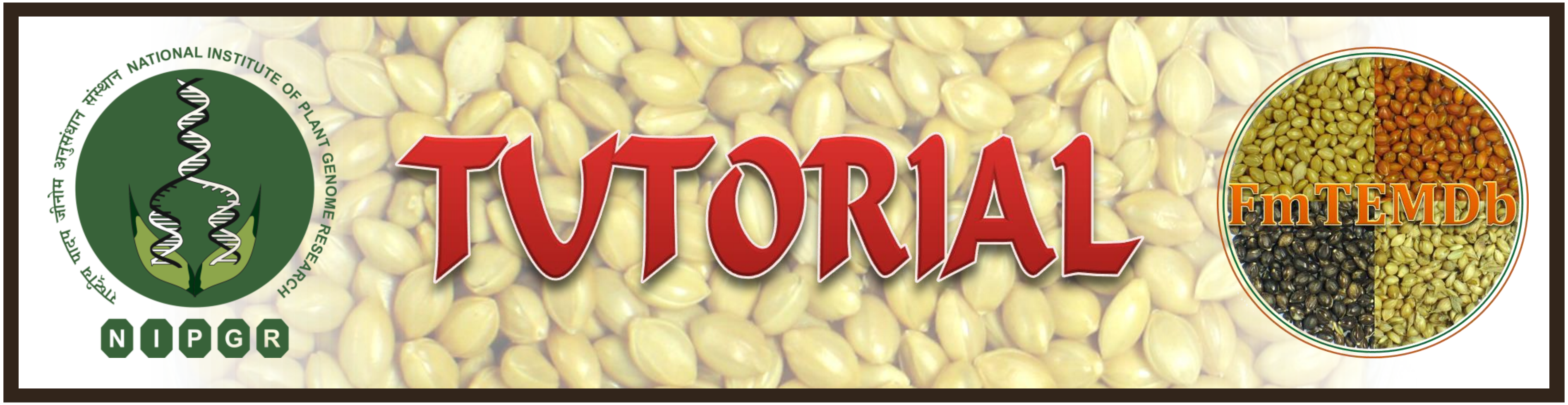
NATIONAL INSTITUTE OF PLANT GENOME RESEARCH
राष्ट्रीय पादप जीनोम अनुसंधान संस्थान
NIPGR
TUTORIAL
FmTEMDb

HOME PAGE

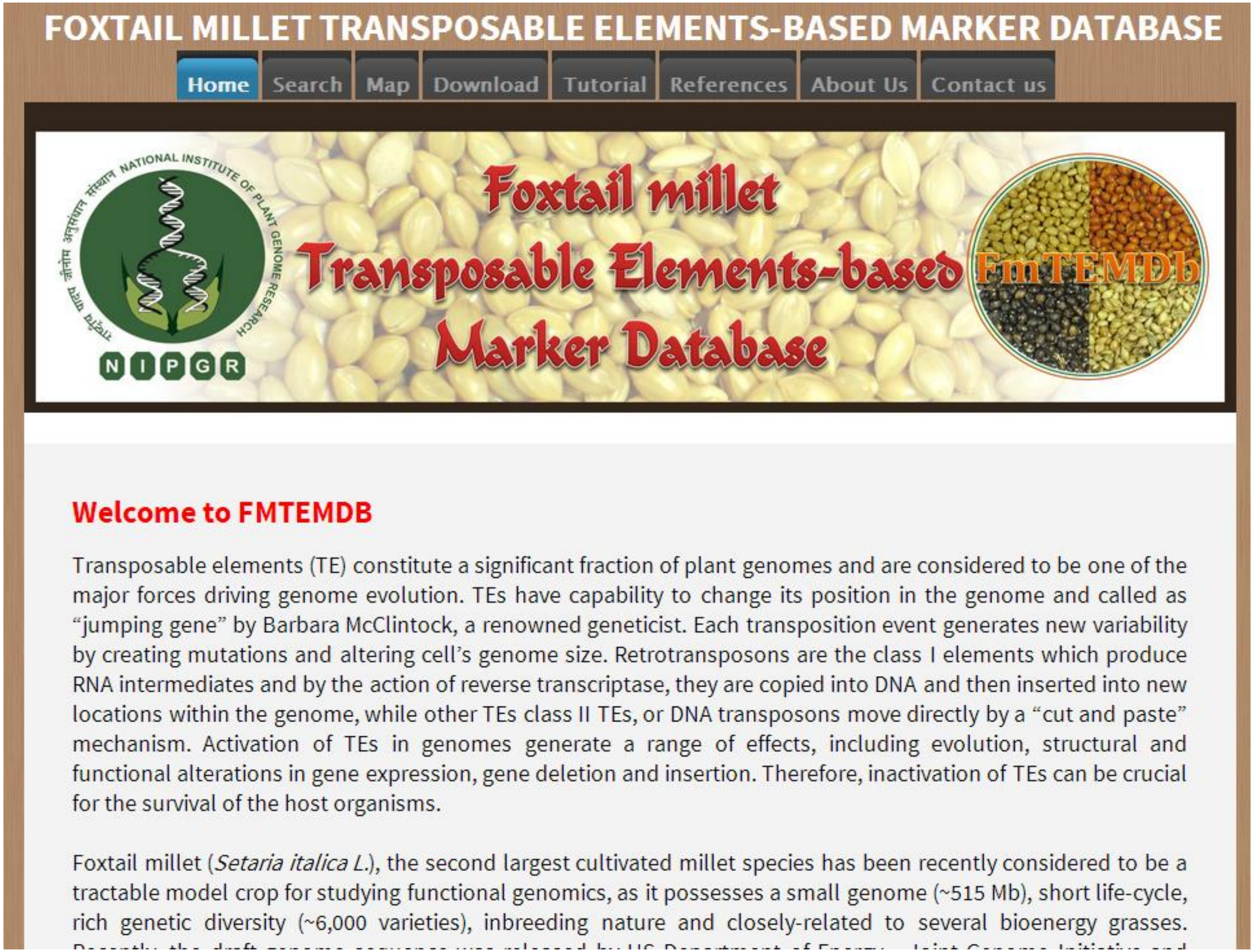

FOXTAIL MILLET TRANSPOSABLE ELEMENTS-BASED MARKER DATABASE

Home | Search | Map | Download | Tutorial | References | About Us | Contact us

Foxtail millet Transposable Elements-based Marker Database

FmTEMDb

Welcome to FMTEMDB

Transposable elements (TE) constitute a significant fraction of plant genomes and are considered to be one of the major forces driving genome evolution. TEs have capability to change its position in the genome and called as "jumping gene" by Barbara McClintock, a renowned geneticist. Each transposition event generates new variability by creating mutations and altering cell's genome size. Retrotransposons are the class I elements which produce RNA intermediates and by the action of reverse transcriptase, they are copied into DNA and then inserted into new locations within the genome, while other TEs class II TEs, or DNA transposons move directly by a "cut and paste" mechanism. Activation of TEs in genomes generate a range of effects, including evolution, structural and functional alterations in gene expression, gene deletion and insertion. Therefore, inactivation of TEs can be crucial for the survival of the host organisms.

Foxtail millet (*Setaria italica L.*), the second largest cultivated millet species has been recently considered to be a tractable model crop for studying functional genomics, as it possesses a small genome (~515 Mb), short life-cycle, rich genetic diversity (~6,000 varieties), inbreeding nature and closely-related to several bioenergy grasses. [illegible]

NAVIGATION BAR

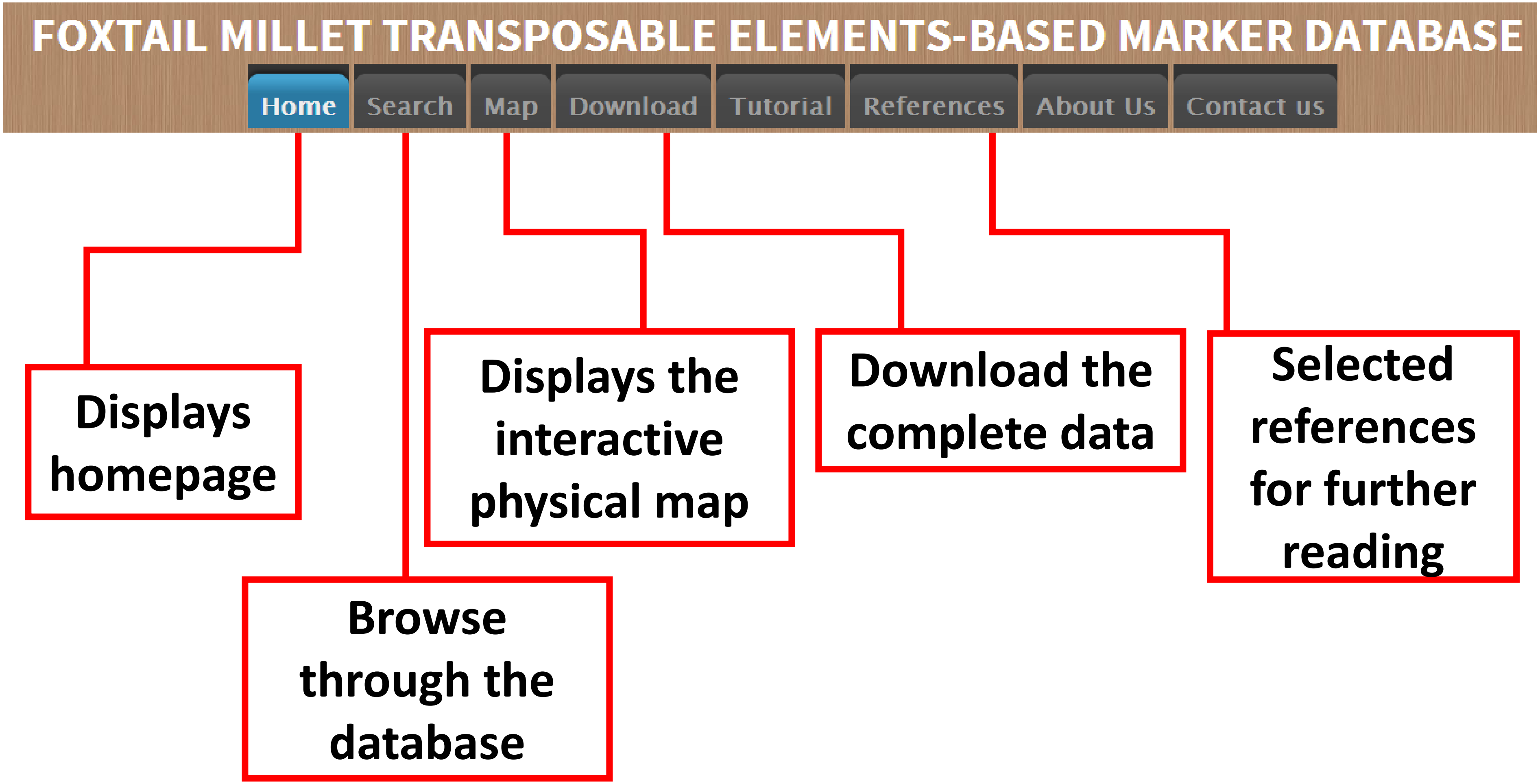

'SEARCH' TAB

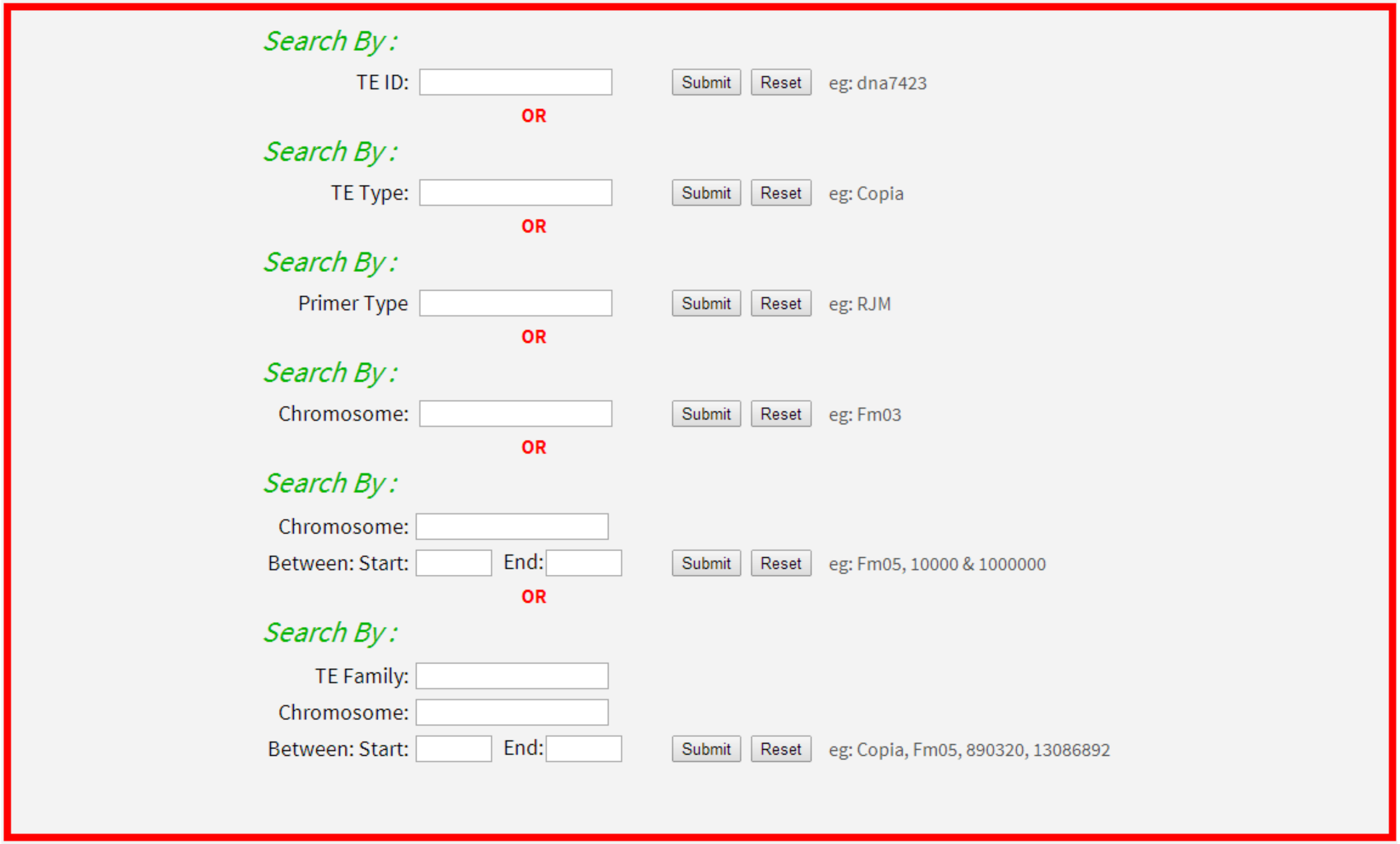

The 'SEARCH' tab provides the user a wide-range of search features including searching by chromosome, type of TE, type of primer, ID of TE, chromosomal location and TE family etc.

'SEARCH BY TE ID'

Search By :

TE ID: dna1039 | Submit | Reset | eg: dna7423

OR

Search By :

TE Type:

Search By :

Primer Type

Search By :

Chromosome: | Submit | Reset | eg: Fm03

OR

Search By :

Chromosome:

Between: Start: | End: | Submit | Reset | eg: Fm05, 10000 & 1000000

OR

Search By :

TE Family:

Chromosome:

Between: Start: | End: | Submit | Reset | eg: Copia, Fm05, 890320, 13086892

Transposon Details:

TE ID	dna7423	TE Type	DNA/CMC-EnSpm
Chromosome	Fm03	Start	50471905
End	50472629	Score	5
Strand	-	Physical Position	View
Get	Primers		

'SEARCH BY TE ID'

Search By :

TE ID: dna1039 Submit Reset eg: dna7423

OR

Search By :

TE Type:

Search By :

Primer Type

Search By :

Chromosome:

OR

Search By :

Chromosome:

Between

Transposon Details:

TE ID	dna7423	TE Type	DNA/CMC-EnSpm
Chromosome	Fm03	Start	50471905
End	50472629	Score	5
Strand	-	Physical Position	View
Get	Primers		

Primers Details: Part-I TE ID: dna1039

Primer Type	Junction	TE Type	TE Source	Orientation	Primer Start	Primer Len	Tm°C	GC%
ISBP	938	DNA transposon-TIR-CACTA, Unknown	trep_complete	FORWARD	899	20	59.93	50

Primers Details: Part-II TE ID: dna1039

Primer Seq	Junctiona_spanning_loc	Insertion_type	Product_size	Primer_type	Junction	TE_Type	TE_source	Orientation
GAGAAACCTTGGTATGCCCA	0	Fragment	317	ISBP	938	DNA transposon-TIR-CACTA, Unknown	trep_complete	REVERSE

Primers Details: Part-III TE ID: dna1039

Start_primer	Primer_Len	Tm°C	GC%	Primer_Seq	Junctiona_spanning_loc2	Insertion_type
1215	20	60.11	45	TCCGTGTAGCAAATGTGGAA	0	Fragment

'SEARCH BY TE ID'

Search By :

TE ID: dna1039 Submit Reset eg: dna7423

OR

Search By :

TE Type:

Search By :

Primer Type

Search By :

Chromosome:

OR

Search By :

Chromosome:

Between

Transposon Details:

TE ID	dna7423	TE Type	DNA/CMC-EnSpm
Chromosome	Fm03	Start	50471905
End	50472629	Score	5
Strand	-	Physical Position	View
Get	Primers		

Primers Details: Part-I TE ID: dna1039

Primer Type	Junction	TE Type	TE Source	Orientation	Primer Start	Primer Len	Tm^{0}C	GC%
ISBP	938	DNA transposon-TIR-CACTA, Unknown	trep_complete	FORWARD	899	20	59.93	50

Primers Details: Part-II TE ID: dna1039

Primer Seq	Junctiona_spanning_loc	Insertion_type	Product_size	Primer_type	Junction	TE_Type	TE_source	Orientation
GAGAAACCTTGGTATGCCCA	0	Fragment	317	ISBP	938	DNA transposon-TIR-CACTA, Unknown	trep_complete	REVERSE

Primers Details: Part-III TE ID: dna1039

Start_primer	Primer_Len	Tm^{0}C	GC%	Primer_Seq	Junctiona_spanning_loc2	Insertion_type
1215	20	60.11	45	TCCGTGTAGCAAATGTGGAA	0	Fragment

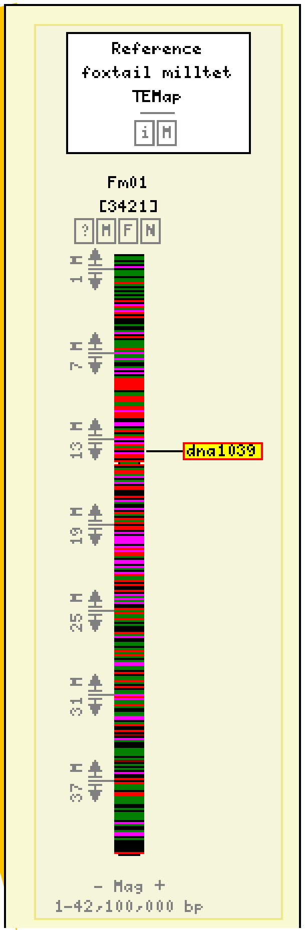

'SEARCH BY TE TYPE'

Search By:

TE ID: [ ] Submit Reset eg: dna7423

OR

Search By:

TE Type: Copia Submit Reset eg: Copia

OR

Search By:

Primer Type [ ] Submit

OR

Search By:

Chromosome: [ ] Submit

OR

Search By:

Chromosome: [ ]

Between: Start: [ ] End: [ ] Submit Reset eg: Fm05, 10000 & 1000000

OR

Search By:

TE Family: [ ]

Chromosome: [ ]

Between: Start: [ ] End: [ ] Submit Reset eg: Copia, Fm05, 890320, 13086892

TE Type: Copia Total TEs found: 1038

TE Type	TE ID	Get
Copia	copia235	More Details
Copia	copia236	More Details
Copia	copia237	More Details
Copia	copia250	More Details
Copia	copia251	More Details
Copia	copia252	More Details
Copia	copia253	More Details
Copia	copia254	More Details
Copia	copia255	More Details
Copia	copia256	More Details
Copia	copia257	More Details

'SEARCH BY TE TYPE'

Search By:

TE ID: Submit Reset eg: dna7423

OR

Search By:

TE Type: Copia Submit Reset eg: Copia

OR

Search By:

Primer Type Submit

OR

Search By:

Chromosome: Submit

OR

Search By:

Chromosome:

Between: Start: End: Submit Reset eg: Fm05 [illegible]

OR

TE Type: Copia Total TEs found: 1038

TE Type	TE ID	Get
Copia	copia235	More Details
Copia	copia236	More Details
Copia	copia237	More Details
Copia	copia250	More Details
Copia	copia251	More Details
Copia	copia252	More Details
Copia	copia253	More Details
Copia	copia254	More Details
Copia	copia255	More Details
Copia	copia256	More Details
Copia	copia257	More Details

Copia Details:

TE ID	copia256	Chromosome	Fm03
Start	16160283	End	16166976
Strand	+	Score	6694
Physical Position	View	LTR Region Similarity	6
5`-LTR Start	2147483647	5`-LTR End	16160283
5`-LTR Length	16160720	3`-LTR Start	438
3`-LTR Length	16166525	3`-LTR Length	16166976
Get	Primers		

‘SEARCH BY TE TYPE’

Copia Details:

TE ID	copia256	Chromosome	[illegible]
Start	16160283	End	[illegible]
Strand	+	Score	[illegible]
Physical Position	View	[illegible]	[illegible]
5`-LTR Start	2147483647	[illegible]	[illegible]
5`-LTR Length	16160720	[illegible]	[illegible]
3`-LTR Length	16166525	[illegible]	[illegible]
Get	Primers		[illegible]

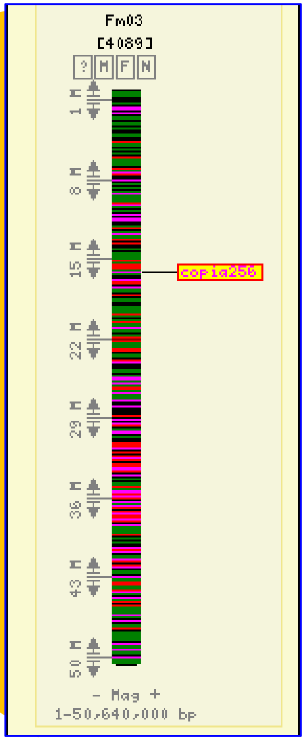

'SEARCH BY TE TYPE'

Copia Details:

TE ID	copia256	Chromosome	[illegible]
Start	16160283	End	[illegible]
Strand	+	Score	[illegible]
Physical Position	View	[illegible]	[illegible]
5`-LTR Start	2147483647	5`-[illegible]	[illegible]
5`-LTR Length	16160720	3`-LTR [illegible]	[illegible]
3`-LTR Length	16166525	3`-LTR Len[illegible]	[illegible]
Get	Primers		

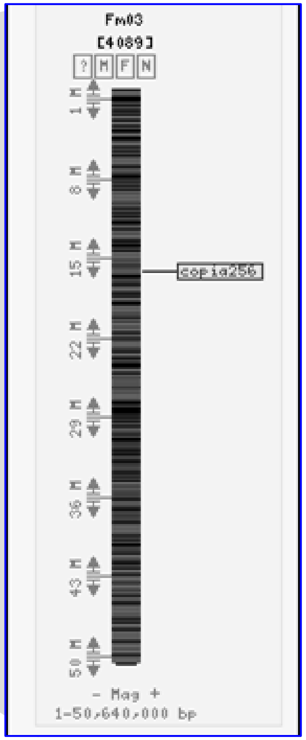

Primers Details: Part-I TE ID: copia256

Primer No	Primer Type	Junction	TE Type	TE Source	Orientation	Primer Start	Primer Len	Tm⁰C	GC%
1	ISBP	2688			FORWARD	2297	20	59.78	50

Primers Details: Part-II TE ID: copia256

Primer Seq	Junctiona_spanning_loc	Insertion_type	Product_size	Primer_type	Junction	TE_Type	TE_source	Orientation
GAAGCCAATGGATCTTGAGC	0	Fragment	470	ISBP	2688			REVERSE

Primers Details: Part-III TE ID: copia256

Start_primer	Primer_Len	Tm⁰C	GC%	Primer_Seq	Junctiona_spanning_loc2	Insertion_type
2766	20	59.88	40	TTGCAACATGAACAGTTGCA	0	Fragment

'SEARCH BY PRIMER TYPE'

Search By :

TE ID: [ ] Submit Reset eg: dna7423

OR

Search By :

TE Type: [ ] Submit Reset eg: Copia

OR

Search By :

Primer Type RJM Submit Reset eg: RJM

OR

Search By :

Primers Type Details: RJM Total Found:712

TE ID	Primer Type	TE Type	Get
copia1000	RJM	Copia	More Details
copia1004	RJM	Copia	More Details
copia1008	RJM	Copia	More Details
copia1009	RJM	Copia	More Details
copia1010	RJM	Copia	More Details
copia1012	RJM	Copia	More Details
copia1013	RJM	Copia	More Details
copia1015	RJM	Copia	More Details

‘SEARCH BY PRIMER TYPE’

Search By :

TE ID: [ ] Submit Reset eg: dna7423

OR

Search By :

TE Type: [ ] Submit Reset eg: Copia

OR

Search By :

Primer Type RJM Submit Reset eg: RJM

OR

Search By :

Primers Type Details: RJM Total Found:712

TE ID	Primer Type	TE Type	Get
copia1000	RJM	Copia	More Details
copia1004	RJM	Copia	[illegible] Details
copia1008	RJM	[illegible]	[illegible] Details
[illegible]	[illegible]	[illegible]	[illegible]

Copia Details:

TE ID	copia1000	Chromosome	Fm09
Start	32740008	End	32748171
Strand	+	Score	8164
Physical Position	View	LTR Region Similarity	6
5`-LTR Start	2147483647	5`-LTR End	32740008
5`-LTR Length	32741408	3`-LTR Start	1401
3`-LTR Length	32746771	3`-LTR Length	32748171
Get	Primers		

'SEARCH BY PRIMER TYPE'

Copia Details:

TE ID	copia1000	Chromosome	[illegible]
Start	32740008	End	[illegible]
Strand	+	Score	[illegible]
Physical Position	View	[illegible]	[illegible]
5`-LTR Start	2147483647	5`-[illegible]	[illegible]
5`-LTR Length	32741408	3`-LTR[illegible]	[illegible]
3`-LTR Length	32746771	3`-LTR Le[illegible]	[illegible]
Get	Primers		

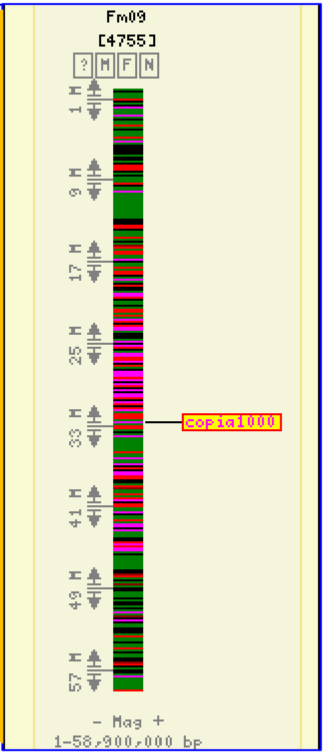

‘SEARCH BY PRIMER TYPE’

Copia Details:

TE ID	copia1000	Chromosome	[illegible]
Start	32740008	End	[illegible]
Strand	+	Score	[illegible]
Physical Position	View	[illegible]	[illegible]
5`-LTR Start	2147483647	5`-[illegible]	[illegible]
5`-LTR Length	32741408	3`-LTR[illegible]	[illegible]
3`-LTR Length	32746771	3`-LTR Le[illegible]	[illegible]
Get	Primers		

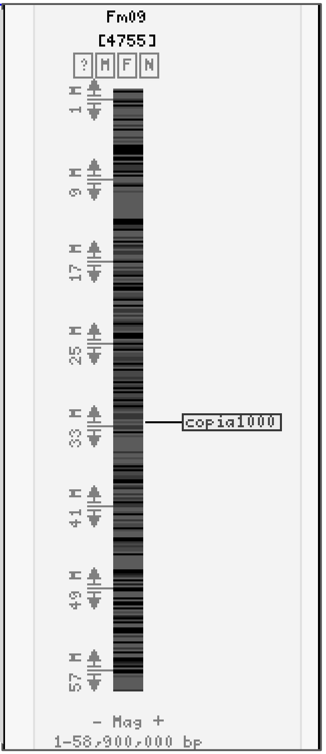

Primers Details: Part-I TE ID: copia1000

Primer No	Primer Type	Junction	TE Type	TE Source	Orientation	Primer Start	Primer Len	Tm^{0}C	GC%
1	RJM	986	Retrotransposon-LTR-Gypsy, Unknown	trep_complete	FORWARD	972	20	62.54	55
2	RJM	1878	Unknown, Retrotransposon-LTR-Copia	trep_complete	FORWARD	1680	20	59.97	50
3	RJM	2848	Unknown, Retrotransposon-LTR-Copia	trep_complete	FORWARD	2193	19	60.76	58
4	RJM	2958	Retrotransposon-LTR-Copia, Retrotransposon-LTR-Copia	trep_complete	FORWARD	2724	20	61.04	55
5	RJM	4036	Retrotransposon-LTR-Copia, Unknown	trep_complete	FORWARD	4021	21	60.04	48
6	RJM	4036	Retrotransposon-LTR-Copia, Retrotransposon-LTR-Copia		FORWARD	3830	20	59.81	50
1	ISBP	986	Retrotransposon-LTR-Gypsy, Unknown	trep_complete	FORWARD	935	20	60.11	50
2	ISBP	1375	Retrotransposon-LTR-Gypsy, Retrotransposon-LTR-Copia	trep_complete	FORWARD	721	20	60.21	50

'SEARCH BY CHROMOSOME'

Search By:

TE ID: [ ] Submit Reset eg: dna7423

OR

Search By:

TE Type: [ ] Submit Reset eg: Copia

OR

Search By:

Primer Type [ ] Submit Reset eg: RJM

OR

Search By:

Chromosome: Fm03 Submit Reset eg: Fm03

OR

Search By:

Chromosome: [ ]

Between: Start: [ ] End: [ ] Submit

OR

Search By:

TE Family: [ ]

Chromosome: [ ]

Between: Start: [ ] End: [ ] Submit

Chromosome: Fm03 Total TEs found: 4089

TE Type	TE ID	Get
Copia	copia235	More Details
Copia	copia236	More Details
Copia	copia237	More Details
Copia	copia250	More Details
Copia	copia251	More Details
Copia	copia252	More Details
Copia	copia253	More Details
Copia	copia254	More Details
Copia	copia255	More Details
Copia	copia256	More Details
Copia	copia257	More Details

'SEARCH BY CHROMOSOME'

Search By:

TE ID: Submit Reset eg: dna7423

OR

Search By:

TE Type: Submit Reset eg: Copia

OR

Search By:

Primer Type Submit Reset eg: RJM

OR

Search By:

Chromosome: Fm03 Submit Reset eg: Fm03

OR

Search By:

Chromosome:

Between: Start: End: Submit

OR

Search By:

TE Family:

Chromosome:

Between: Start: End: Submit

Chromosome: Fm03 Total TEs found: 4089

TE Type	TE ID	Get
Copia	copia235	More Details
Copia	copia236	More Details
Copia	copia237	More Details
Copia	copia250	More Details
Copia	copia251	More Details
Copia	copia252	More Details
Copia	copia253	More Details
Copia	copia254	More Details
Copia	copia255	More Details
Copia	copia256	More Details
Copia	[illegible]	[illegible]

Copia Details:

TE ID	copia256	Chromosome	Fm03
Start	16160283	End	16166976
Strand	+	Score	6694
Physical Position	View	LTR Region Similarity	6
5\`-LTR Start	2147483647	5\`-LTR End	16160283
5\`-LTR Length	16160720	3\`-LTR Start	438
3\`-LTR Length	16166525	3\`-LTR Length	16166976
Get	Primers		

‘SEARCH BY CHROMOSOME’

Copia Details:

TE ID	copia256	Chromosome	Fm03
Start	16160283	End	[illegible]
Strand	+	Score	[illegible]
Physical Position	View	[illegible]	[illegible]
5`-LTR Start	2147483647	[illegible]	[illegible]
5`-LTR Length	16160720	[illegible]	[illegible]
3`-LTR Length	16166525	[illegible]	[illegible]
Get	Primers		

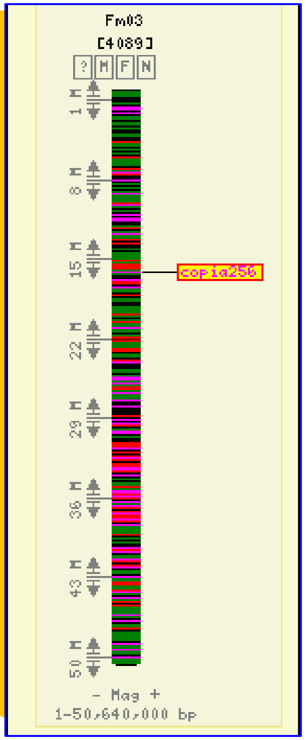

'SEARCH BY PRIMER TYPE'

Copia Details:

TE ID	copia1000	Chromosome	[illegible]
Start	32740008	End	[illegible]
Strand	+	Score	[illegible]
Physical Position	View	[illegible]	[illegible]
5`-LTR Start	2147483647	5`-[illegible]	[illegible]
5`-LTR Length	32741408	3`-LTR[illegible]	[illegible]
3`-LTR Length	32746771	3`-LTR Le[illegible]	[illegible]
Get	Primers		

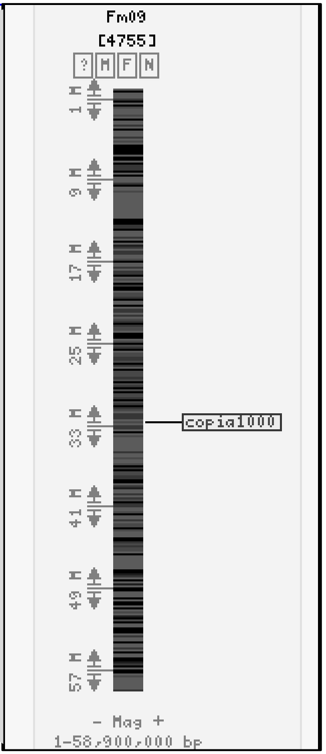

Primers Details: Part-I TE ID: copia1000

Primer No	Primer Type	Junction	TE Type	TE Source	Orientation	Primer Start	Primer Len	Tm^{0}C	GC%
1	RJM	986	Retrotransposon-LTR-Gypsy, Unknown	trep_complete	FORWARD	972	20	62.54	55
2	RJM	1878	Unknown, Retrotransposon-LTR-Copia	trep_complete	FORWARD	1680	20	59.97	50
3	RJM	2848	Unknown, Retrotransposon-LTR-Copia	trep_complete	FORWARD	2193	19	60.76	58
4	RJM	2958	Retrotransposon-LTR-Copia, Retrotransposon-LTR-Copia	trep_complete	FORWARD	2724	20	61.04	55
5	RJM	4036	Retrotransposon-LTR-Copia, Unknown	trep_complete	FORWARD	4021	21	60.04	48
6	RJM	4036	Retrotransposon-LTR-Copia, Retrotransposon-LTR-Copia		FORWARD	3830	20	59.81	50
1	ISBP	986	Retrotransposon-LTR-Gypsy, Unknown	trep_complete	FORWARD	935	20	60.11	50
2	ISBP	1375	Retrotransposon-LTR-Gypsy, Retrotransposon-LTR-Copia	trep_complete	FORWARD	721	20	60.21	50

‘MAP’ OPTION

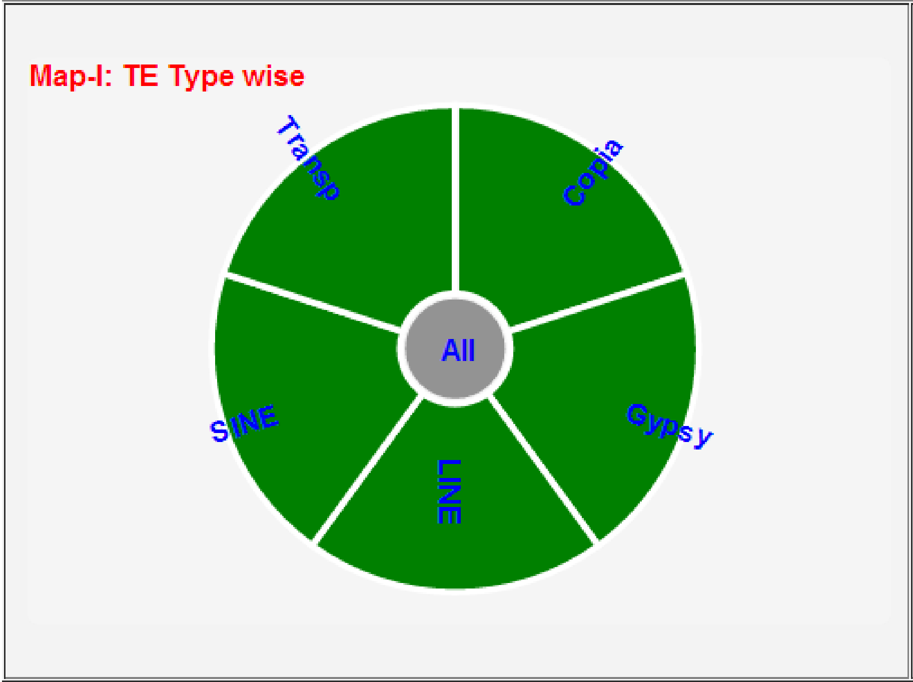
Map-I: TE Type wise
Transp
Copia
All
SINE
Gypsy
LINE

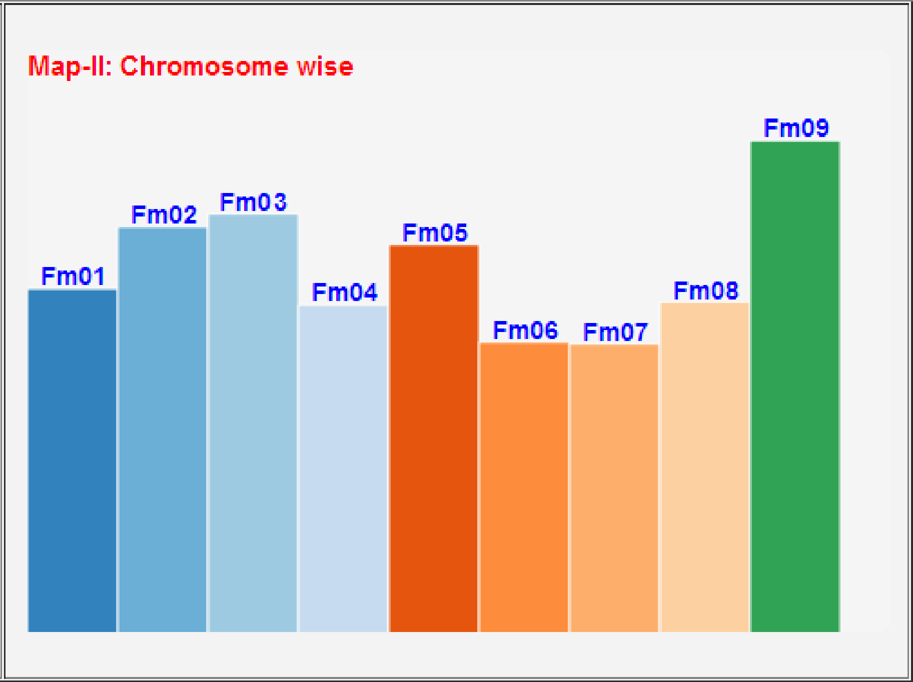
Map-II: Chromosome wise
Fm01
Fm02
Fm03
Fm04
Fm05
Fm06
Fm07
Fm08
Fm09

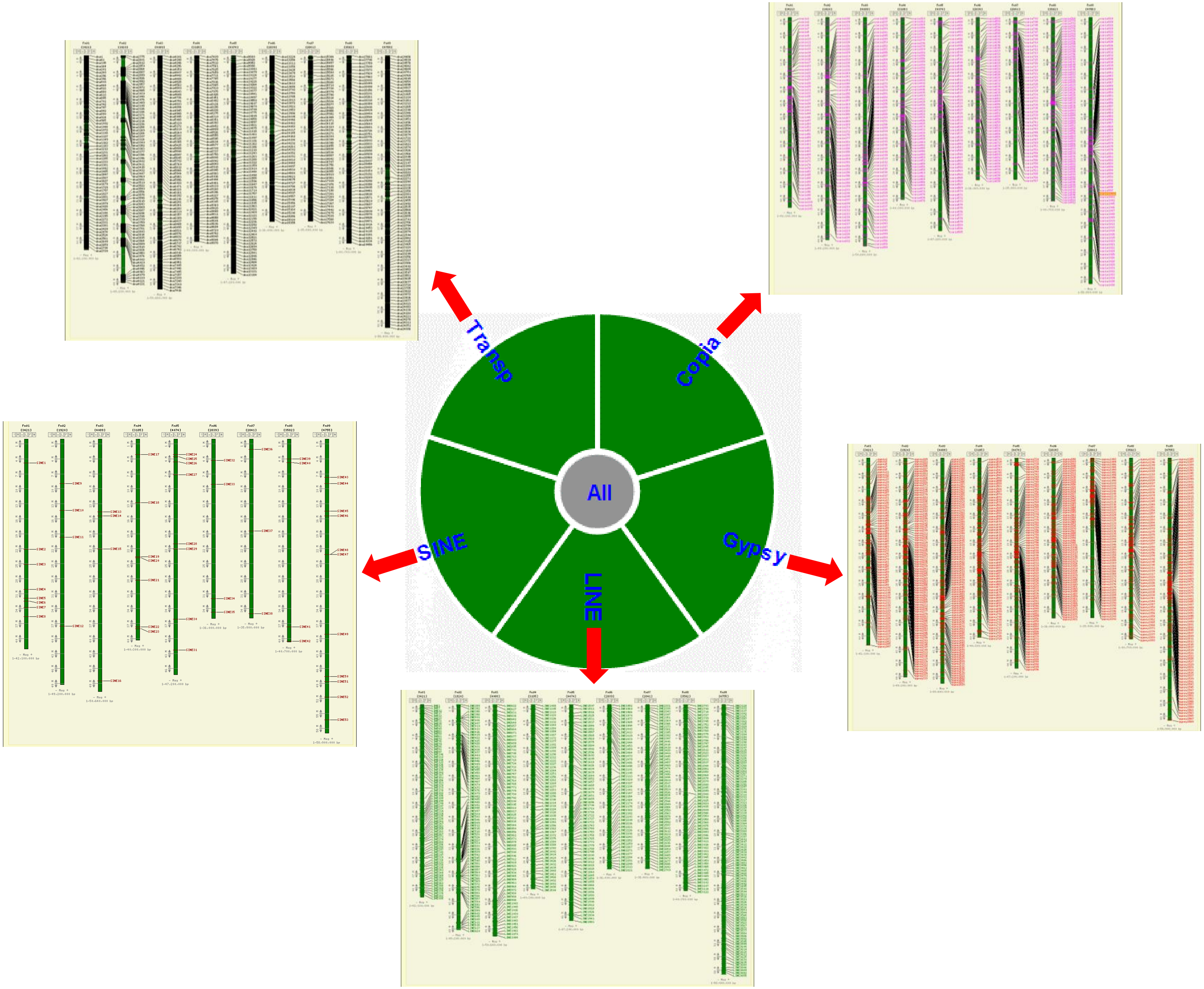
Transp
Copia
All
SINE
Gypsy
LINE

HOW TO VIEW THE DETAILS

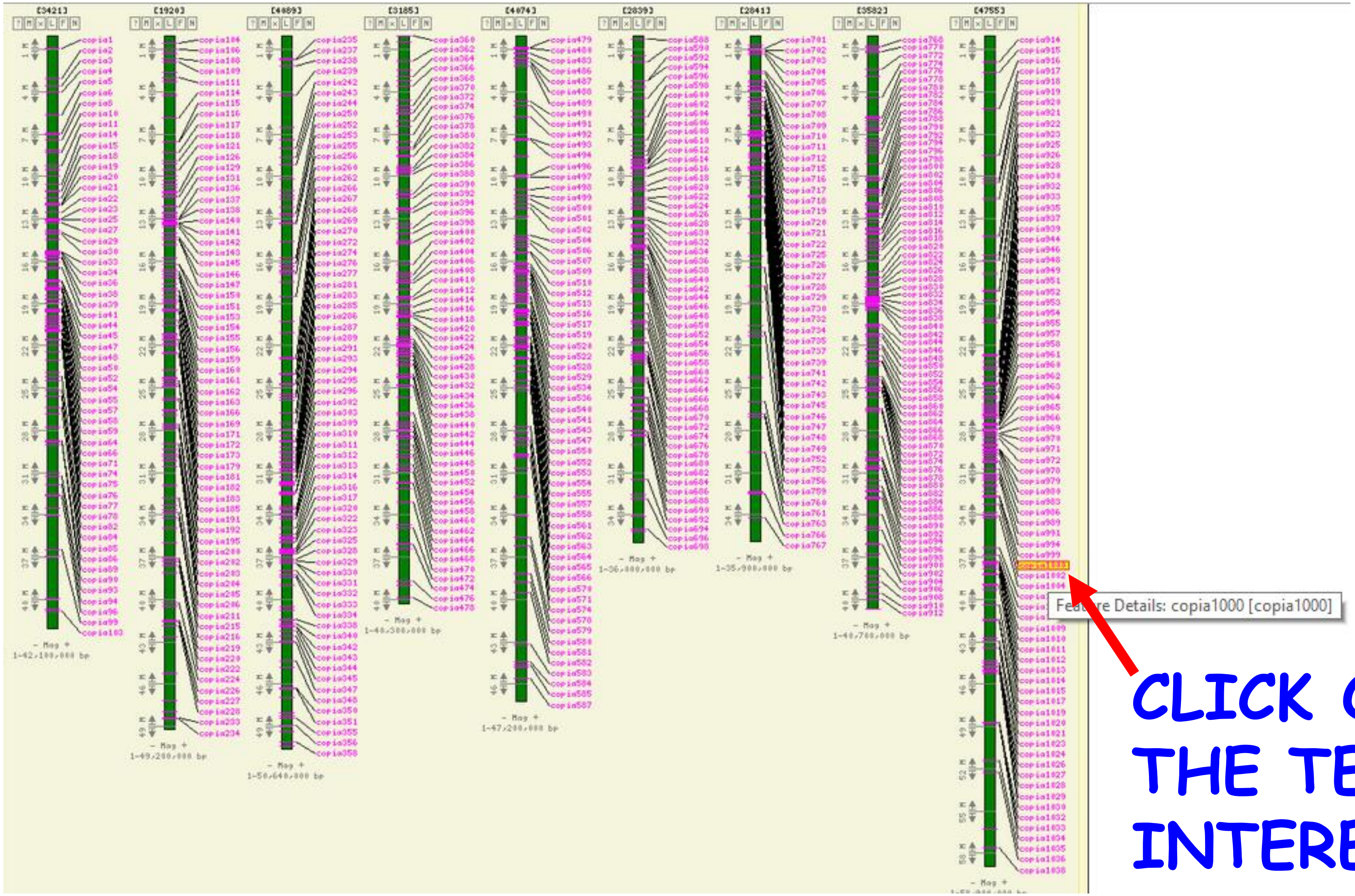

CLICK ON THE TE OF INTEREST

HOW TO VIEW THE DETAILS

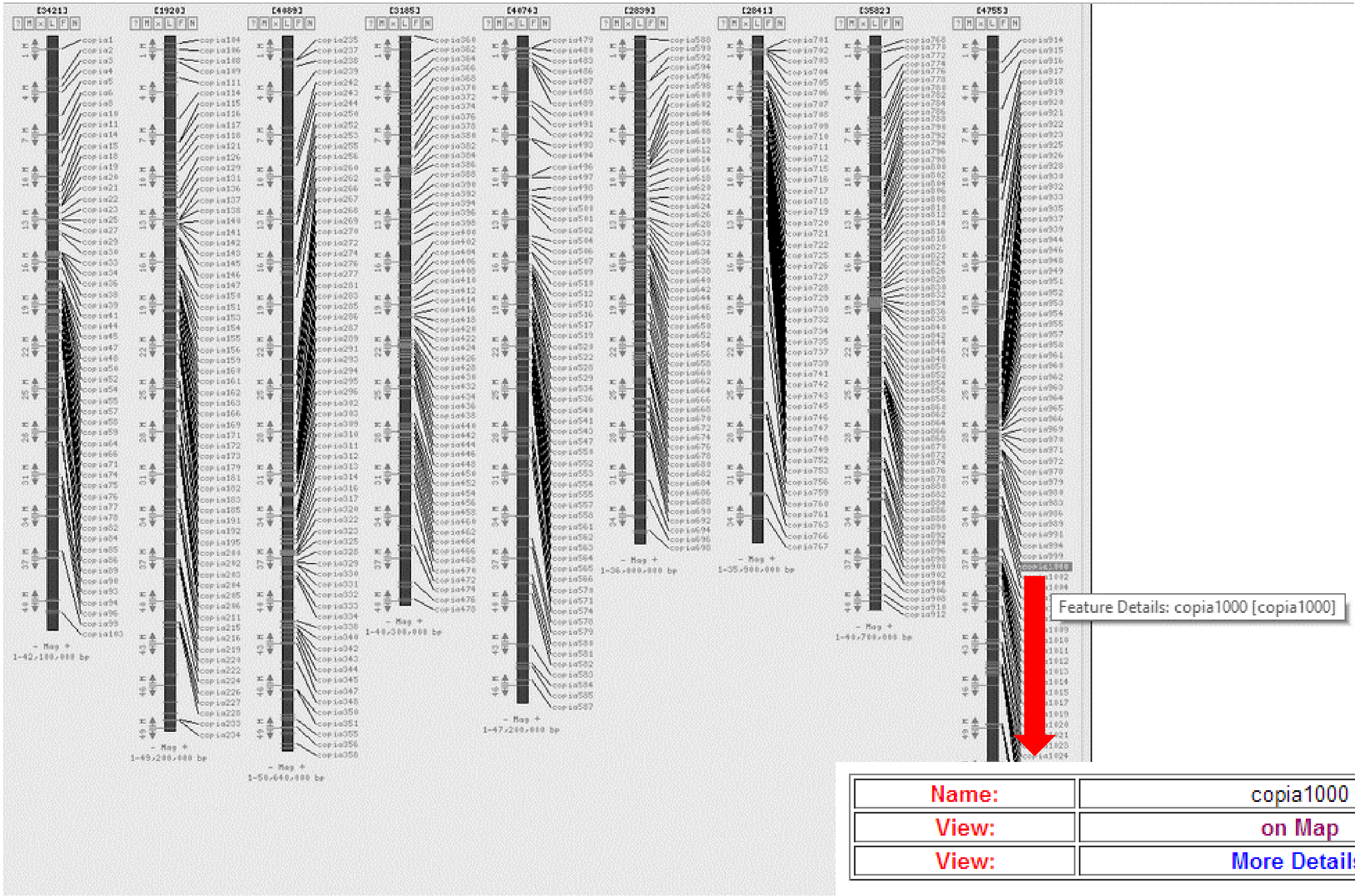

Name:	copia1000
View:	on Map
View:	More Details

VIEWING THE DETAILS

Name:	copia1000
View:	on Map
View:	More Details

Copia Details:

TE ID	copia1000	Chromosome	[illegible]
Start	32740008	End	[illegible]
Strand	+	Score	[illegible]
Physical Position	View	[illegible]	[illegible]
5`-LTR Start	2147483647	5`-[illegible]	[illegible]
5`-LTR Length	32741408	3`-LTR[illegible]	[illegible]
3`-LTR Length	32746771	3`-LTR Le[illegible]	[illegible]
Get	Primers		

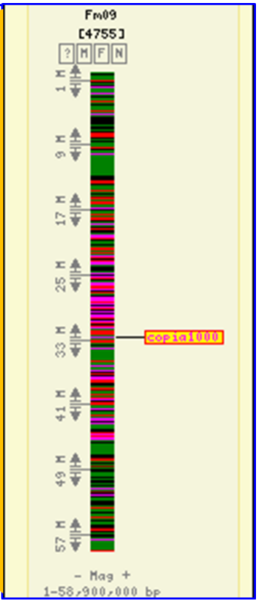

Primers Details: Part-I TE ID: copia1000

Primer No	Primer Type	Junction	TE Type	TE Source	Orientation	Primer Start	Primer Len	Tm^{0}C	GC%
1	RJM	986	Retrotransposon-LTR-Gypsy, Unknown	trep_complete	FORWARD	972	20	62.54	55
2	RJM	1878	Unknown, Retrotransposon-LTR-Copia	trep_complete	FORWARD	1680	20	59.97	50
3	RJM	2848	Unknown, Retrotransposon-LTR-Copia	trep_complete	FORWARD	2193	19	60.76	58
4	RJM	2958	Retrotransposon-LTR-Copia, Retrotransposon-LTR-Copia	trep_complete	FORWARD	2724	20	61.04	55
5	RJM	4036	Retrotransposon-LTR-Copia, Unknown	trep_complete	FORWARD	4021	21	60.04	48
6	RJM	4036	Retrotransposon-LTR-Copia, Retrotransposon-LTR-Copia		FORWARD	3830	20	59.81	50
1	ISBP	986	Retrotransposon-LTR-Gypsy, Unknown	trep_complete	FORWARD	935	20	60.11	50
2	ISBP	1375	Retrotransposon-LTR-Gypsy, Retrotransposon-LTR-Copia	trep_complete	FORWARD	721	20	60.21	50

Chromosome-wise Map

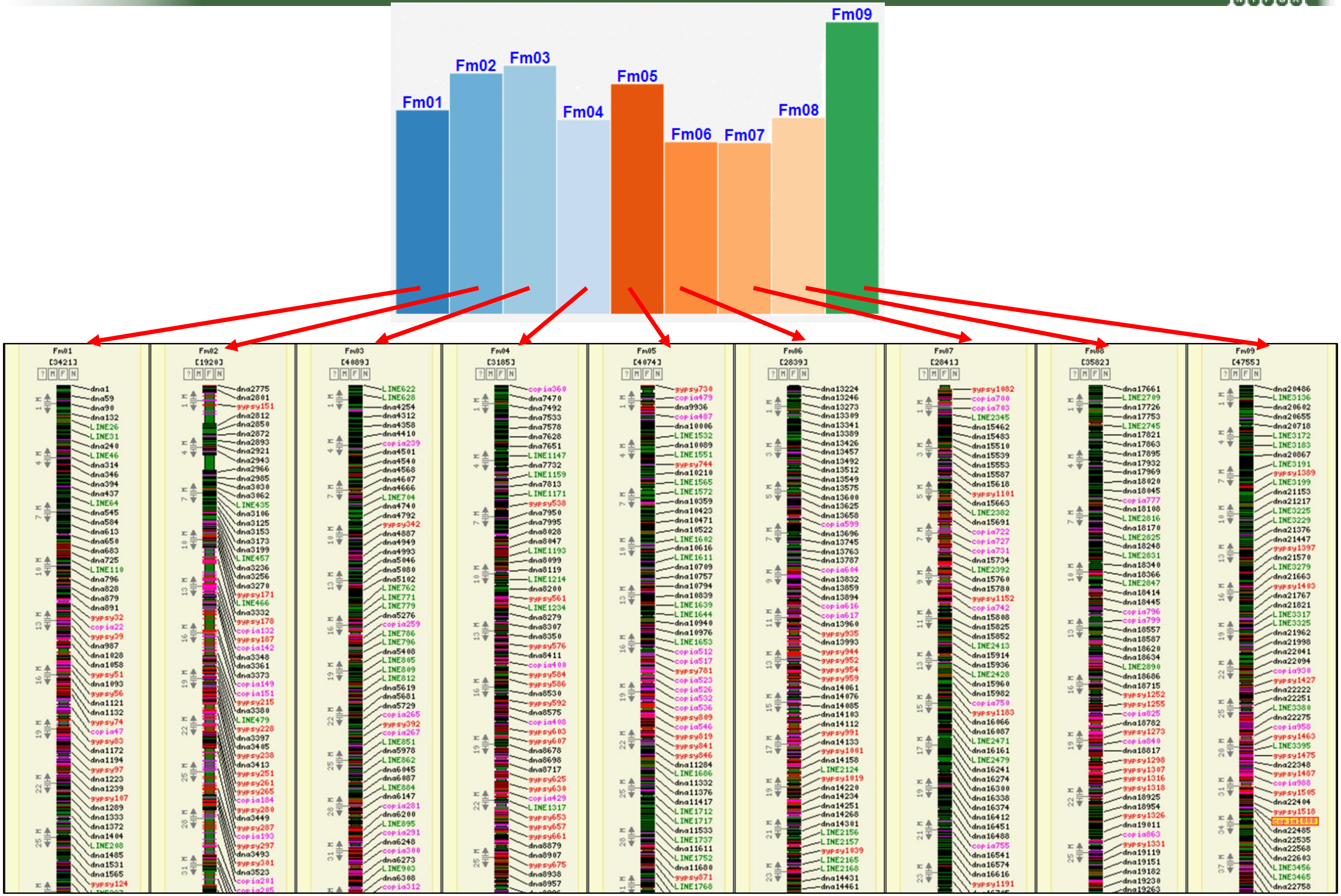

Comparative map

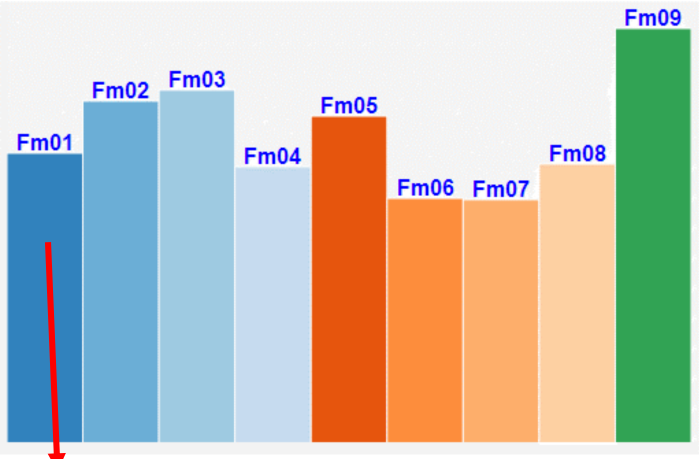

TE-marker based comparative mapping data is available for visualization with sorghum, maize, rice and *Brachypodium*

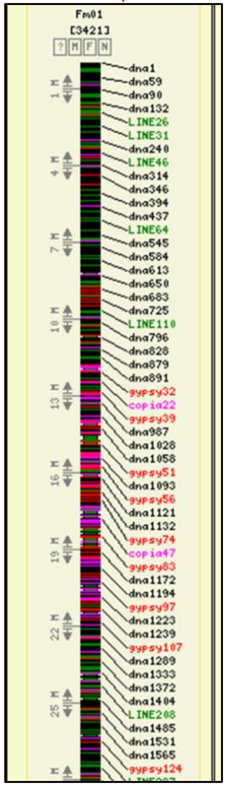

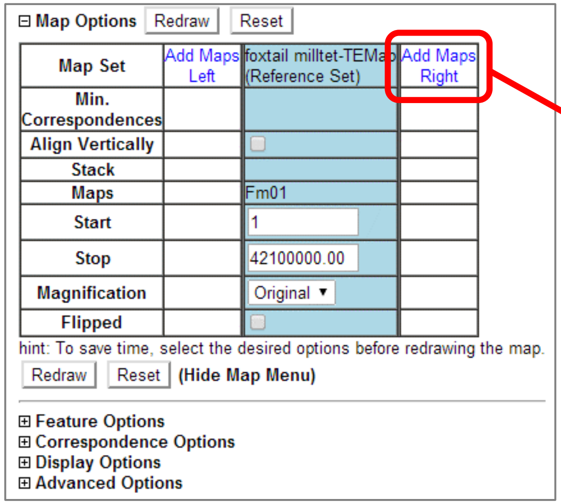

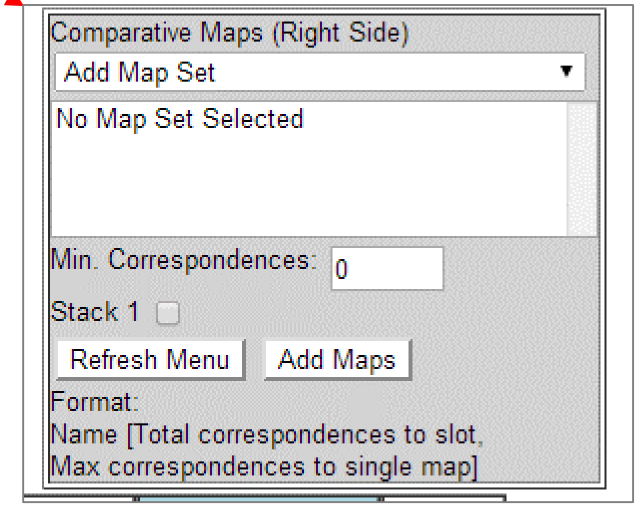

Comparative map

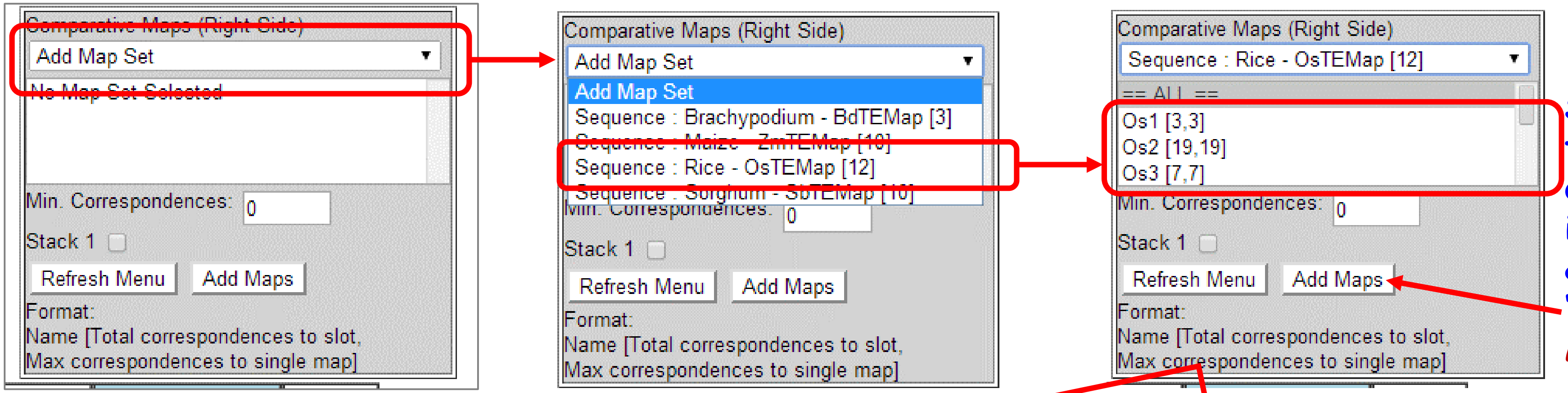

Select the chr. of interest & click 'Add Maps'

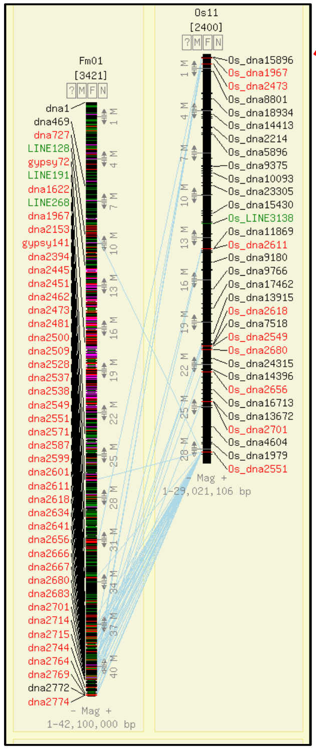

The Syntenic TEs are displayed in RED and the blue lines indicate the orthologs

Download Data

The complete TE & marker data of foxtail millet are available for download in this section.

Download Data

Type of TE	TE Data	Primers
Copia	Download	Download
Gypsy	Download	Download
LINE	Download	No Primers
SINE	Download	No Primers
Transposon	Download	Download
