# Supplementary material for: Genome-wide development of transposable elements-based markers in foxtail millet and construction of an integrated database
Source: DNA Res. 2014 Nov 26;22(1):79–90. doi: 10.1093/dnares/dsu039 (PMC4379977; doi:10.1093/dnares/dsu039)
Supplement: Supplementary Data [file supp_dsu039_dsu039supp_table24.doc]

**Supplementary Table S24:** Determination of K using Evanno’s method

| **K** | **Reps** | **Mean LnP(K)** | **Stdev LnP(K)** | **Ln'(K)** | **|Ln''(K)|** | **Delta K** |
| --- | --- | --- | --- | --- | --- | --- |
| 2 | 3 | -1217.23 | 0.3055 | NA | NA | NA |
| 3 | 3 | -1070.9 | 2.0075 | 146.3333 | 0.266667 | 0.132836 |
| 4 | 3 | -924.3 | 0.5292 | 146.6 | 62.36667 | 117.8619 |
| 5 | 3 | -840.067 | 15.1533 | 84.23333 | 3.266667 | 0.215574 |
| 6 | 3 | -759.1 | 20.0592 | 80.96667 | 3.966667 | 0.197748 |
| 7 | 3 | -682.1 | 0.3464 | 77 | 37.43333 | 108.0607 |
| 8 | 3 | -642.533 | 11.1715 | 39.56667 | 1.166667 | 0.104432 |
| 9 | 3 | -604.133 | 19.1126 | 38.4 | 7.766667 | 0.406363 |
| 10 | 3 | -573.5 | 3.8118 | 30.63333 | 12.46667 | 3.270526 |
| 11 | 3 | -555.333 | 2.2502 | 18.16667 | 0.733333 | 0.325899 |
| 12 | 3 | -536.433 | 1.6042 | 18.9 | NA | NA |
